# Speciated mechanism in Quaternary cervids (*Cervus* and *Capreolus*) on both sides of the Pyrenees: a multidisciplinary approach

**DOI:** 10.1038/s41598-022-24684-7

**Published:** 2022-11-23

**Authors:** Antigone Uzunidis, Anna Rufà, Ruth Blasco, Jordi Rosell, Jean-Philip Brugal, Pierre-Jean Texier, Florent Rivals

**Affiliations:** 1 Institut Català de Paleoecologia Humana I Evolució Social (IPHES-CERCA), Zona Educacional 4, Campus Sescelades URV (Edifici W3), 43007 Tarragona, Spain; 2 ICArEHB – Interdisciplinary Centre for Archaeology and the Evolution of Human Behaviour, Universidade Do Algarve, Campus de Gambelas, 8005-139 Faro, Portugal; 3 Univ. Bordeaux, CNRS, MCC, PACEA, UMR 5199, F-33600 Pessac, France; 4 Departament d’Història I Història de L’Art, Universitat Rovirai Virgili, Av. Catalunya 35, 43002 Tarragona, Spain; 5 CNRS, Aix-Marseille Université, Minist. Cult., UMR 7269 LAMPEA, F13097 Aix-en-Provence Cedex 2, France; 6 ICREA, Pg. Lluís Companys 23, 08010 Barcelona, Spain

**Keywords:** Palaeoecology, Palaeontology

## Abstract

Cervids, and especially the red deer *Cervus elaphus*, are among the most regularly and abundantly recorded ungulates in Pleistocene/Paleolithic bone assemblages. Numerous Pleistocene or Holocene subspecies have been described, reinforcing their status as essential proxies for environmental and chronological reconstructions. Despite this, at the beginning of the Late Pleistocene, their diversity seems to have decreased. In this study, we analysed teeth and some postcranial elements of *Cervus* and *Capreolus* from north-eastern Iberia and south-eastern France to clarify their morphological characteristics and ecological adaptations. We describe a transitional form in north-eastern Iberia between the western European stock and the current form *C. e. hispanicus*. Such sub-speciation processes are connected to biogeographical factors, as there were limited exchanges between north-eastern Iberia and the northern Pyrenees, whereas the north-western part of the peninsula seems more connected to the northern Pyrenees. The anatomical plasticity (morpho-functional adaptation and body size) of red deer is connected to dietary flexibility (dental meso- and microwear). Conversely, *Capreolus* shows greater morphological and ecological homogeneity. Body size variations seem directly correlated with their ability to browse throughout the year. The marked differences between the eco-bio-geographical responses of the two taxa can be explained by their habitat selection.

## Introduction

Cervids are among the most common herbivores in the Mediterranean Pleistocene. Many genera have adapted or even specialised to this region: *Cervus*, *Capreolus*, *Dama*, *Megaloceros*, *Megaceroides* and *Haploidoceros* (excluding Villafranchian forms). *Capreolus* and *Cervus* are more often present in the archaeological and palaeontological record in western Europe, the former much less abundant than the latter. They are therefore particularly useful taxa for biochronological and palaeoenvironmental reconstructions.

Deer are capable of adapting to a wide variety of environments and biotopes across a very large area of the northern hemisphere. This adaptability is linked to their great morphological plasticity, which has led to the description of numerous species and subspecies^1–3^. *Cervus simplicidens* was erected from MIS 5 archaeofaunal remains of Combe Grenal (Dordogne, France) based on its small body size and the morphology of the fourth premolars and third lower molars^4,5^. It has been recognised in several sites in southern France and northern Italy^5–9^. This species is not widely accepted^10^, however, and after a revision of the holotype material, Croitor^11^ refuted the existence of this species, believing that it could be integrated into the variability of *Cervus elaphus*. Since the Late Pleistocene, the majority of Western European deer remains are thus ascribed to *Cervus elaphus*. Several red deer subspecies are identified in Western Europe^12^: *C. e. elaphus*, *C. e. atlanticus* (Norway), *C. e. scoticus* (Scotland), *C. e. corsicanus* (Corsica and Sardinia) and *C. e. hispanicus* (Spain). According to genetic studies, the differentiation of these subspecies dates from the Last Glacial Maximum^13^, or predates it^14,15^. The plastic diversity of Quaternary red deer has been linked to their ability to occupy diverse environments, particularly due to their varied diet. They can feed on woody and non-woody plants, grasses and even fruits^16–18^. Nevertheless, the mechanisms governing deer body adaptations, including body mass, are complex, and may depend on climatic conditions^19^, paleogeography^20^ or dietary resources^21^.

Several species of roe deer have been described since the Middle Pleistocene, but only *Capreolus capreolus* is known in the Late Pleistocene^3^, and no subspecies has been described. Several subspecies of roe deer are currently known, however, notably *C. c. garganta* in Spain and *C. c. italicus* in Italy^22,23^. Genetic analyses suggest that the Iberian populations separated from the European pool between isotope stages 5 and 3, and the Italian populations during the LGM^24^. Compared to red deer, roe deer are much more selective in their feeding, and when fruits and seeds are available, they select them or switch to a more browser-based diet^25^. Seasonally, they may also consume grasses^26^ but mostly remain typical browsers^17,19^. Variations in body mass known from the fossil record seem to be mainly chronological^3^, but not related to major climatic events^19^. Size variations have been observed in current populations, and connected to several ecological parameters^27^.

While genetic studies find evidence of speciation as early as MIS 3 (ca. 57–24 kyrs), palaeontological studies do not reflect this emerging diversity. In this case, does the absence of Late Pleistocene subspecies reflect a time of homogenisation in the skeleton morphology favoured by the admixture of cervid populations? Or was the period between the last two glacial maximums (MIS 6 and 2), despite its great climatic instability, a period of homogenisation in the ecological preferences of *Cervus* and *Capreolus* respectively?

This paper aims to analyse the morphological characteristics and ecological adaptations on cervids during the MIS 4 to 3 in north-eastern Iberia by combining different proxies. To this end, we will focus on two archaeological sites from south-eastern France (Pié Lombard) and north-eastern Spain (Teixoneres). They are located in refugia areas^28,29^ that have facilitated the survival and the maintenance of temperate taxa such as *Cervus* and *Capreolus* even during cold periods. Despite being subjected to the same favourable Mediterranean climate, local environmental conditions were different (see below) raising the question of the ecological adaptations of these two taxa. Moreover, the two sites are located on both side of the Gulf of Lion in a small but fragmented geographical area. Several topographic barriers segment this area, in particular the Rhône River to the east and the Pyrenean Mountain range to the west, which marks the boundary between the current red deer and roe deer subspecies. During the Late Pleistocene, however, the presence of cold species in the peninsula suggests at least, a periodical passage^30^, raising the question of the importance of the impracticable nature of this barrier. The study of the morphometric variability of red and roe deer can be a strong indicator of regular exchanges or population isolation north and south of the Pyrenees and therefore, be indicative of topographical limitations encountered by cervid populations.

## Archaeological sites

### Pié Lombard

**Figure 1:**
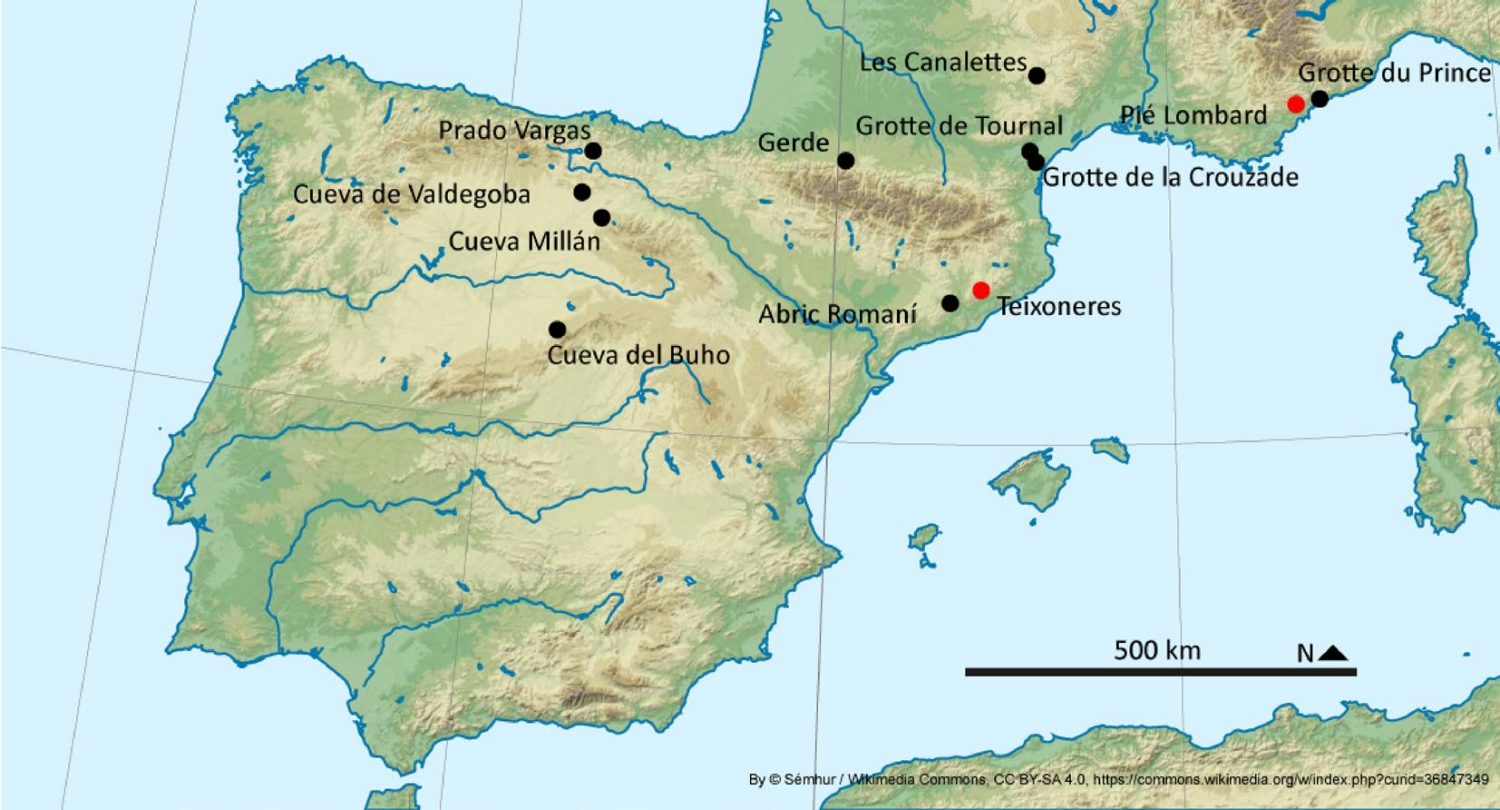
Geographical position of Pié Lombard rockshelter and Teixoneres cave (in red) and of MIS 5 to 3 *Cervus* and *Capreolus* populations used as examples in this work (in black). Map base by Sémhur / Wikimedia Commons, CC BY-SA 4.0, https://commons.wikimedia.org/w/index.php?curid=36847349.

The Pié Lombard site is located near the town of Tourrettes-sur-Loup (Alpes-Maritimes, France) (Fig. 1). It is a very small rockshelter (10 m^2^) located at the foot of a rather steep cliff at a relatively low altitude: 250 m above the sea level (Supplementary Fig. S1). It is located in a very rugged and varied area framed by the Mediterranean Sea 12 km to the south, the Loup River to the east and to the north, and very close mountainous areas culminating in more than 1000 m (Texier et al.,^33^). The site was discovered in 1962^31^, and excavated from 1971 to 1996 (with interruption) under the direction of Pierre-Jean Texier^32,33^. They uncovered lithic and faunal remains accumulated in a ditch at the front of the rockshelter, preserving an archaeological sequence three meters thick.

Several sedimentary levels were recognised in the sequence and it was separated into two main stratigraphic complexes, or *ensembles*^33^:

*Ensemble* I was dated by accelerator mass spectrometry (AMS) to between 20,639 and 20,167 cal BP or 22,581–22,073 cal BP^34^, attributed to the Epipaleolithic culture, yielded very few faunal remains, and is not relevant to this study (for more details see Pelletier et al., 2019^35^).

*Ensemble* II is a Mousterian complex dated to a transitional phase between MIS 5 and 4 based on a thermoluminescence (TL) date of 70 ± 8 kyr BP^36^. Stalagmites supporting Mousterian sediments yielded two electron spin resonance dating (ESR) dates of 147 ± 10 kyr and 130 ± 20 kyr BP^37^.

Sixteen species of large mammals were found in the *Ensemble* II. The best represented species of herbivores were *Capra ibex* and *Cervus elaphus* followed by *Rupicapra rupicapra* and *Capreolus capreolus*, completed by the very scarce remains of *Bos primigenius*, *Equus caballus* and *Sus scrofa*^33^. There are also diverse carnivores at the site, with *Ursus arctos*, *Felis silvestris*, *Lynx* sp., *Panthera pardus* and *Canis lupus*, and some remains of *Vulpes vulpes* and *Cuon* sp.. In addition to this large fauna, small game is exceptionally well represented, with at least 225 rabbits (*Oryctolagus cuniculus*) and various bird species represented by at least 86 individuals (*Alectoris graeca*, *Perdrix perdrix*, *Columbia livia*, *Pyrrhocorax graculus*, *Pyrrhocorax pyrrhocorax*). The small game often bears traces of human activity to retrieve meat, skin and feathers^35,38^. Waterside taxa (e.g. *Castor fiber*, *Arvicola amphibus*) are rare, related to the vicinity of the river. The zooarchaeological analysis suggests the accumulation was mainly due to human activity, especially concerning small game, *Cervus* and *Capra*^33,35,38^, and, to a lesser extent, to carnivores. Finally, two Neanderthal deciduous incisors were also found^33,39^.

The Mousterian lithic industry included 902 pieces made of various materials (flint, siliceous limestone, rhyolite, quartzite, jasper). Their origin was mostly local or semi-local, although some pieces were from further away (about 70 km)^40,41^. The lithic industry was generally introduced to the site as finished tools (e.g. *scrapers*), suggesting hunting activities as the main purpose of Neanderthal occupation at Pié Lombard^41^, which is interpreted as a seasonal hunting camp^42^.

Palynological, faunal and malacofaunal proxies allow to describe the palaeoenvironment of the Pié Lombard Mousterian levels. The climate must have been dry, and cooler than the current climate, although with a Mediterranean trend. Palynological analysis indicates an open environment (Arboreal Pollen between 5 and 9%) while the microfauna indicates the existence of wooded areas^43^. All the environmental proxies for Pié Lombard depict diverse biotopes connected to the diversity of landforms (river side, cliff, mountains, plateaux).

### Teixoneres

Teixoneres cave is located near the village of Moià (Barcelona, Spain), and is part of the Toll Caves karst complex (Fig. 1). The site is located at 780 m a.s.l., within a homogenous countryside (in comparison with Pié Lombard) (Supplementary Fig. S1). The landscape is formed of small hills and small minor streams, with the exception of the Llobregat river in the south and the Ter river in the north, which border the area^44^.

The Toll karstic complex was discovered in the 1950s by a local speleological group. Several excavation seasons were carried out by different research teams until the 1990s. Since 2003, Teixoneres cave has been excavated by a team from the *Institut Català de Paleoecologia Humana i Evolució Social* (IPHES-CERCA)^45–48^.

The cave yielded a six-meter deep sedimentary sequence in which eight stratigraphic units were identified and separated into sub-units^45,46,49^. Units I and IV are speleothems that frame the levels involved in this study (unit II and III). They have been dated by U-series: Unit I has an age of ca. 14–16 ka and unit IV, ca. 100.3 ± 6.1 ka^50^. Unit II has been dated by radiocarbon to 44,210 to 33,060 cal BP^51^ and separated into two subunits: IIa and IIb. Unit III was radiocarbon dated from > 51,000 ^14^C BP to 44,210 cal BP^51^ and was also divided into two subunits: IIIa and IIIb.

The same faunal spectrum is present in the two units, dominated by *Cervus elaphus*, followed by *Equus caballus*, *Equus hydruntinus*, *Bos primigenius*, *Capreolus capreolus*, *Capra pyrenaica*, *Rupicapra pyrenaica* and *Sus scrofa*. Some rare remains of *Coelodonta antiquitatis* and *Mammuthus primigenius* are also reported^44,52^.

In addition to herbivores, carnivores were also found (*Ursus spelaeus*, *Crocuta crocuta*, *Canis lupus*, *Vulpes vulpes*, *Lynx spelaea*, *Meles meles*). Previous studies concluded that humans occupied the cave during short events in alternation with carnivore occupations^44,53–56^. They occupied different parts of the cave: the Neanderthals occupied the entrance, and the other predators occupied the inner parts^47,49^.

Large herbivore mammals at the site display traces that can be attributed to Neanderthal and carnivore predation. The presence of small prey (leporid and bird remains), however, seems mainly due to predator activities^53,54^. Finally, three deciduous human teeth and the molar of an adult were found in Unit IIIb.

The lithic tools from Teixoneres Cave correspond to a Mousterian industry with raw materials of local or semi-local origin. Most of the raw materials are quartz, chert, quartzite, and schist. The majority of the artefacts correspond to completed tools or flakes interpreted as a combination of hunting tools and cutting objects used during the early phases of butchery^57^.

As in Pié Lombard, several methods based on different proxies were applied in Teixoneres Cave to reconstruct the environment. In Unit IIIb, the climate was unstable, slightly warmer than in the other levels, and humid^58^. The environment was dominated by oak-pine forest (AP = 60%)^59^ with the presence of open areas^60,61^. Units IIIa and IIa show strong convergences, both marked by a cool climate with heavy precipitation^58^. The forest is expanding in Unit IIIa, reaching 87% of the AP^59^, but the climate remains unstable. It becomes more stable in Unit II. Unit IIb is still cool but much drier than the other units^58,60^. The forest is reduced to its lowest level in Teixoneres with 58–74% AP^59^. The environment thus remained highly forested. In Unit IIa precipitation increased again^58^, and tree pollens exceeded 65% of the spectrum^59^.

Although there are many oscillations, the climate in Teixoneres thus stays globally cool and humid, allowing the extension of a large forest, contrasting with the dry and more open conditions at Pié Lombard.

## Material and methods

### Material

The fossil remains of *Cervus* and *Capreolus* are very unequal between the different units of Teixoneres and Pié Lombard. Indeed, a MNI was established from the teeth; overall 50 specimens are present in Teixoneres (IIa = 3, IIb = 3, IIIa = 8 and IIIb = 36) and 15 in Pié Lombard. Roe deer are poorly represented in Pié Lombard, with only two individuals, and 10 in Teixoneres (IIb = 1, IIIa = 1 and IIIb = 8).

The bone and dental elements of *Cervus* and *Capreolus* are unevenly represented for the two sites (Supplementary Table S1). The post-cranial elements at Teixoneres are in general heavily broken, which greatly limits observations and comparisons. For these reasons, we have chosen to concentrate the paleontological study on selected bone and dental elements according to their frequencies in all the levels/sites concerned. We focus on the lower teeth (noted by the lower case followed by its number, in opposition to the upper teeth noted by the upper case followed by its number, e.g. second lower molar = m2; second upper molar = M2), in particular the second and third molars, as well as the anterior and posterior third phalanges and the talus.

In order to focus on anatomical characters that are perfectly fixed in the different populations, we have chosen to consider only elements from full adult individuals.

In order to appreciate the eco-biometrical variability of *Cervus* and *Capreolus*, we compare our fossil material to 10 sub-contemporary sites (i.e., Late Pleistocene) spread over four geographical regions: South-East France, the North Pyrenees, South-West Pyrenees and South-East Pyrenees (Fig. 1; Supplementary Table S1). In addition to the fossil records, the analysis will be completed with 10 modern red deer individuals from Spain ascribed to the subspecies *Cervus elaphus hispanicus* (Supplementary Table S2), as a first morphometrical description of this subspecies.

We discarded teeth belonging to very old or very young individuals for the palaeodietary reconstruction, using dental meso- and microwear^62,63^. We took taphonomic alteration into consideration by discarding teeth with broken cuspids in mesowear and those with badly preserved enamel and taphonomic defects in microwear, following the description of King et al.^64^ and Uzunidis et al.^65^. In both cases, we sampled all teeth from third premolars to third molars. Second premolars were never sampled, following the recommendations of Kaiser and Solounias^66^ and Xafis et al.^67^.

The collection from Teixoneres Cave and the modern *C. e. hispanicus* individuals are stored in the IPHES-CERCA in Tarragona, Spain, and the Pié Lombard collection is located in UMR 7269 LAMPEA at the MMSH, Aix-Marseille University, France.

### Methods

Exploring the speciation and adaptation of *Cervus* and *Capreolus* on limited geographical and temporal scales implies the combination of several proxies reflecting differences in both the anatomy and the ecology of cervids. We will use various descriptors: linear morphometrics, body mass estimations, morphological descriptions, geometric morphometrics, and dental wear analyses. Such various combined approaches have never been realised on fossil ungulates, but will allow us to better characterise the fossil cervid populations of Pié Lombard and Teixoneres, and compare them to sub-contemporaneous populations. The compendium of the bones and teeth used in this study for each type of analysis is available in Supplementary Table S3.

#### Linear morphometrics and body mass estimations

The series of *Cervus* and *Capreolus* from Teixoneres and Pié Lombard, and those published for northern Spain and southern France dated at the beginning of the Late Pleistocene, were compared metrically using different methods in order to explore their body size and proportions. Measurements were taken using a digital calliper as illustrated in Supplementary Fig. S2. The teeth were always measured at the base of the crown in order to avoid differences related to the use-wear stage. The mean and standard deviation values were calculated for each sample except when there were less than three individuals.

The talus was the most common bone in the different units of Teixoneres and Pié Lombard, so it was chosen to analyse the differences in size between the populations. Its dimensions (lateral length x distal width) were compared using scatter plots. Where possible, raw data rather than means and standard deviations were used in order to appreciate the variability of sexual dimorphism which may be noticeable in red deer^16^. The relative size of each population, described through the talus measurements, was also compared to those obtained on the lower teeth which does not allow to take into account sexual dimorphism but which allows to consider a larger sample of individuals. The dimensions of the lower teeth were compared using the log-size-index^68,69^ (LSI), represented as a Simpson’s ratio diagram^70^. The means of the metric data from each population were compared to the mean of teeth measurements for *Rangifer tarandus* from Jaurens^71^.

Comparable bone and dental remains of *Capreolus* were not present in any of the levels at Teixoneres or Pié Lombard. In order to compare the body sizes of *Capreolus* with each other, despite this, we chose to estimate their weights using equations^72^. The areas of the upper second molars and lower second and third molars taken at the collar were used to reconstruct the body mass of the roe deer in Teixoneres, Pié Lombard, Les Canalettes, Valdegoga and Gerde.

#### Morphological descriptions

The teeth were also analysed based on qualitative criteria to complete the anatomical description of the *Cervus* and *Capreolus* from Pié Lombard and Teixoneres. The anatomical characteristics recorded have already been discussed by several authors^2,9,11,71,73,74^ and allow qualitative criteria to be considered.

For red deer, we focused on the m2 present in all layers at Teixoneres and Pié Lombard. The sample included 31 m2: 15 from Teixoneres (Unit IIa = 1; IIb = 2; IIIa = 1; IIIb = 11), 6 from Pié Lombard and 10 from the current *C. e. hispanicus*.

The presence or absence of eight morphological criteria on the m2 were observed for Teixoneres, Pié Lombard and *C. e. hispanicus*, and used for a MCA analysis performed in R v. 4.2-Rstudio v4.1.3 (function *mca*). Projections of the metaconid, entoconid, parastylid, metastylid and entostylid pillars were used, as well the presence of the ectostylid, anterior fold and at least a cingulum. To identify clusters of related populations, we used the Rstudio function *hclust* to perform a hierarchical agglomerative clustering (HAC) with the Ward’s minimum variance method using the squared Euclidian distance and the first three components of the MCA (72.27% expressed). The optimal number of clusters minimising total intra-cluster variation was determined using a distance criterion and the elbow method.

#### Geometric morphometrics

Geometric morphometric (GMM) is a quantitative approach which allows the comparison of bone shapes and the visualisation of morphological changes between groups. GMM captures morphological variations in skeletal elements that may correspond to various processes such as domestication^75–77^, speciation^78,79^ and ecomorphological adaptations^80,81^. We took this approach on two distinct elements in this study: the m3 and the third phalanges, selecting only the complete elements.

The lower third molars had a very strong phylogenetic signal^5^, confirmed by GMM in Bovini^82^ or Caprini^83^. GMM analysis is not yet applied to red deer teeth, unlike the more traditional morphometric studies, which are much more numerous. Here, the combination of these two approaches will allow us to control and reinforce the results of the analysis carried out on a small number of remains. The sample includes 6 m3 from Pié Lombard, 5 from Teixoneres (IIIa = 2; IIIb = 3) and 3 from *C. e. hispanicus*. In order to avoid morphological variations related to the specimen age, we selected only teeth in the early stages of wear, but with the entire occlusal surface erased, including the third lobe (the most numerous case). We digitised 11 landmarks and 30 semilandmarks between Landmarks 7 and 8 in two dimensions on each m3 (Supplementary Fig. S3) The landmarks are positioned inside the inner edge of the enamel (except for the Landmarks 7 and 8) to the points of maximum curvatures, and the semilandmarks were used to characterise the contours of the third lobe on the exterior edge of the enamel (including Landmarks 7 and 8) which was chosen in this case because of its better reproducibility rate.

GMM had already been applied on the cervids third phalanx, especially the plantar margin, and demonstrates their strong ecomorphofunctional adaption to the type of soil-substrate^80,81^. It is therefore like the diet, an adaptation to local environmental conditions. The distinction was made between anterior and posterior phalanges following the criteria from Herrera (1990)^84^ and Pfeiffer (1999)^85^. The sample included 7 anterior phalanges (6 from Pié Lombard and 1 from Teixoneres Unit IIb) and 6 posterior phalanges (5 from Pié Lombard and 1 from Teixoneres Unit IIIb). We digitised 5 landmarks and 5 curves (Supplementary Fig. S3): between Landmarks 1 and 2 (12 semilandmarks), between Landmarks 2 and 3 (5 semilandmarks), between Landmarks 3 and 4 (10 semilandmarks), between Landmarks 4 and 5 (20 semilandmarks) and between Landmarks 5 and 1 (10 semilandmarks). All landmarks and semilandmarks are positioned on the outside edge of the plantar margin of the third phalange. Allometry was assessed using multivariate regressions of shape variables on the log-transformed centroid sizes. The landmarks and semilandmarks acquisition were realised using the tpsDig2 v. 2.17 software^86^. We performed a generalised Procrustes analysis^87^ (GPA) using Rstudio with the *procSym* function in Morpho R package v. 2.0.3^88^ to analyse shape among the samples. The centroid size^89^ represents the individual size of specimens, and its variation in the considered taxa is shown by boxplots. Principal component analysis (PCA) was performed on the Procrustes shape variables to identify the orthogonal axes of maximal variation in the data set.

#### Dental wear analysis

We performed meso- and microwear analysis on the teeth in order to reconstruct their diet and to explore ecological variability among fossil *Cervus* and *Capreolus* from Pié Lombard and Teixoneres.

*Mesowear analysis* is a method based on the observation of wear patterns on ungulate molar cusps that indicate the abrasiveness of the diet of an individual animal^62,63^. The sharpness and the morphology of the tips of the cusp are correlated with relative attritive and abrasive dental wear. A diet with low abrasion (and high attrition) thus shows very sharp molar buccal cusps, and, conversely, a diet with high abrasion will result in rounded and blunted cusps. It is unclear which time period of the animal’s life is reflected in the teeth mesowear: weekly, seasonal, yearly or even its lifetime^63,90–96^.

This study employed the standardised method proposed by Mihlbachler et al.^97^ and modified by Rivals et al.^98^, which is already used in other works^99–101^. This method categorises dental wear into seven groups (numbered from 0 to 6), according to their shape (from 0 = high and sharp; to 6 = blunt with no relief). The average value of mesowear data from a single sample corresponds to the “mesowear score” (MWS)^97^.

The *microwear analysis* study follows the protocol established by Solounias and Semprebon^102^ and Semprebon et al.^103^. The occlusal surface of each tooth was cleaned using acetone and then 96% alcohol. The surface was then moulded with a high-resolution silicone (vinylpolysiloxane), and casts were made using clear epoxy resin. The transparent casts were then examined with a stereomicroscope at magnifications of × 35. Observations were restricted to a standard surface of 0.16 mm^2^ (using an ocular reticule), preferably localised on the upper tooth paracone and the lower tooth protoconid.

Microwear is understood to record diet over the last days to months of an individual’s life^104–106^. The micro-traces, scratches and pits are left on the occlusal surfaces during mastication^107^. The variability in the density of these traces due to the presence of phytoliths in the plants is indicative of various diets: grazer, mixed-feeder and browser. We observed various features following the classification of Solounias and Semprebon^102^ and Semprebon et al.^103^: pits (small and large), scratches (fine, coarse and hypercoarse), and gouges. A scratch width score (SWS) was also calculated, with a score of ‘0’ for teeth with predominantly fine scratches per tooth surface, ‘1’ for those with mixed fine and coarse scratches on the tooth surface, and ‘2’ for those with predominantly coarse scratches.

## Results

### Morphological characteristics of *Cervus *and *Capreolus *from Pié Lombard, Teixoneres and *Cervus e. hispanicus* teeth

The upper teeth of the Teixoneres and Pié Lombard red deer show similar morphological characteristics. The entostyle is almost always present and often quite high, especially on M2 in Teixoneres and M3 in Pié Lombard (Supplementary Table S4). On molars, the lingual cingulum is also almost always present, whereas it is uncommon in *C. e. hispanicus*. On the other hand, the jugal styles are often poorly expressed in Teixoneres, whereas they are prominent in Pié Lombard and in *C. e. hispanicus*. When a protocone fold is present, it is also more regularly positioned on the posterior part of the fossa than on the anterior part, as is the case in Pié Lombard.

**Figure 2:**
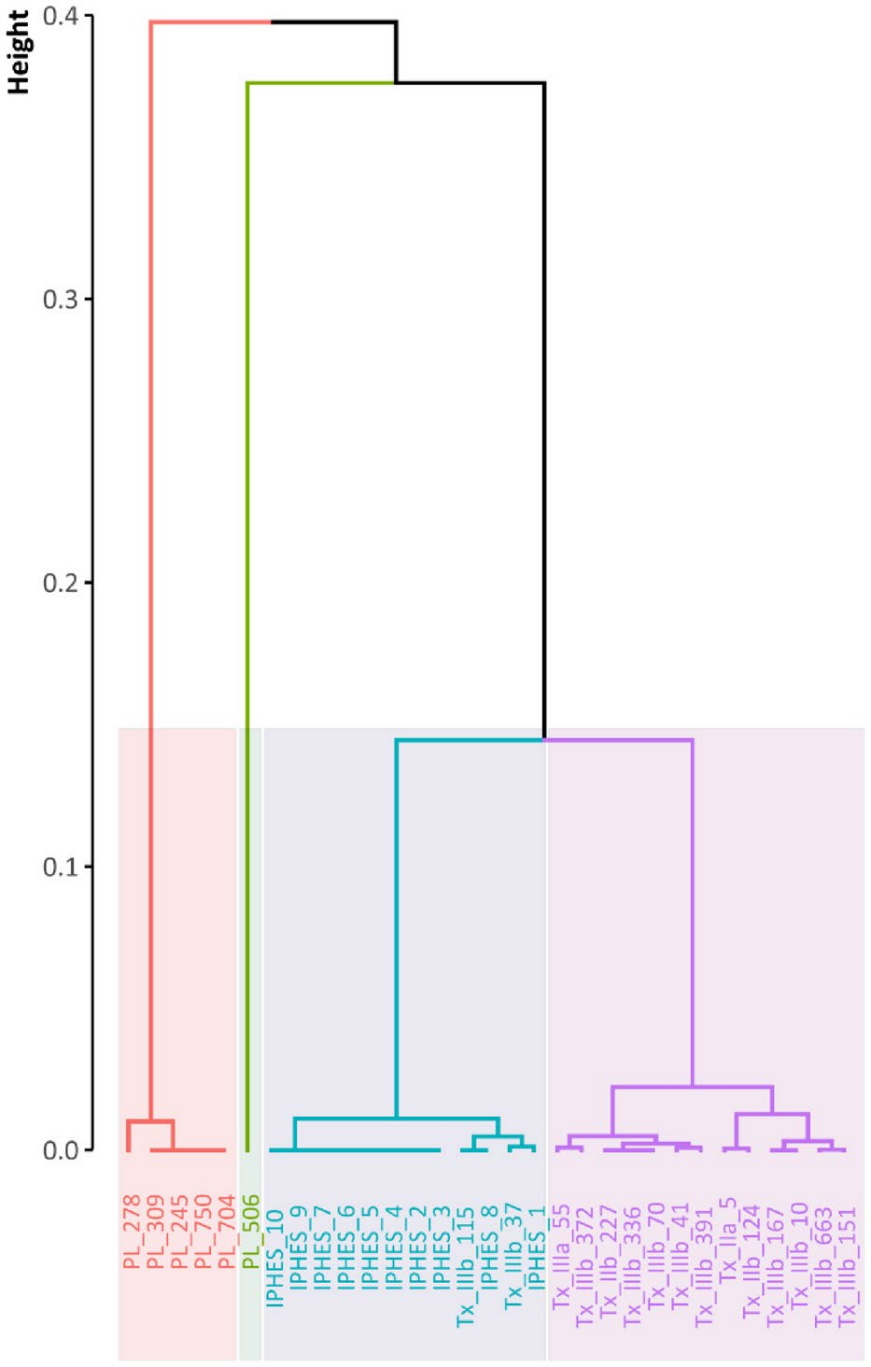
Cluster analysis (Ward’s method) of the first three components of the MCA describing the presence/absenceof *Cervus* m2’s morphological parameters (see Supplementary Table S6). The identifiers are the name of the sites (PL = Pié Lombard; IPHES = *C. e. hispanicus*; Tx = Teixoneres) followed by the name of the unit (when relevant) and the number of the specimen.

Morphological differences between the fossils are most marked on the lower teeth. In general, the ectostylid is present, well expressed and increasing in size from m1 to m3 in Teixoneres, whereas an opposite pattern can be observed in Pié Lombard (Supplementary Table S5). In general, p4 are weakly “molarised” at Teixoneres: the metaconid was rarely fused to the paraconid and the entoconid to the entostylid (20% of p4 in Unit IIIa and 30% of p4 in Unit IIIb). At Pié Lombard, this feature is much more common (66% of p4), and is the majority for *C. e. hispanicus* (71% of p4). The relevance of this criterion in the study of cervid evolution is considered unreliable, however^11,108,109^. Pillars and styles are very often poorly developed on lower molars from Teixoneres. The lower molars are much simpler than those of Pié Lombard (Supplementary Table S6). Statistical comparison using MCA and hierarchical clustering of morphological characters of the m2 within the series of Pié Lombard, Teixoneres and *C. e. hispanicus* clearly discriminates the four groups (Fig. 2). The m2s from Teixoneres red deer from all units show common features that isolate them from the current *C. e. hispanicus*. The m2s of the latter are also isolated in a group and mixed with two teeth from the unit IIIb of Teixoneres which could be related with the morphological variability in a deer population. At last, the m2s from Pié Lombard are split in two groups that are quite distinct from the Iberian populations.

**Figure 3:**
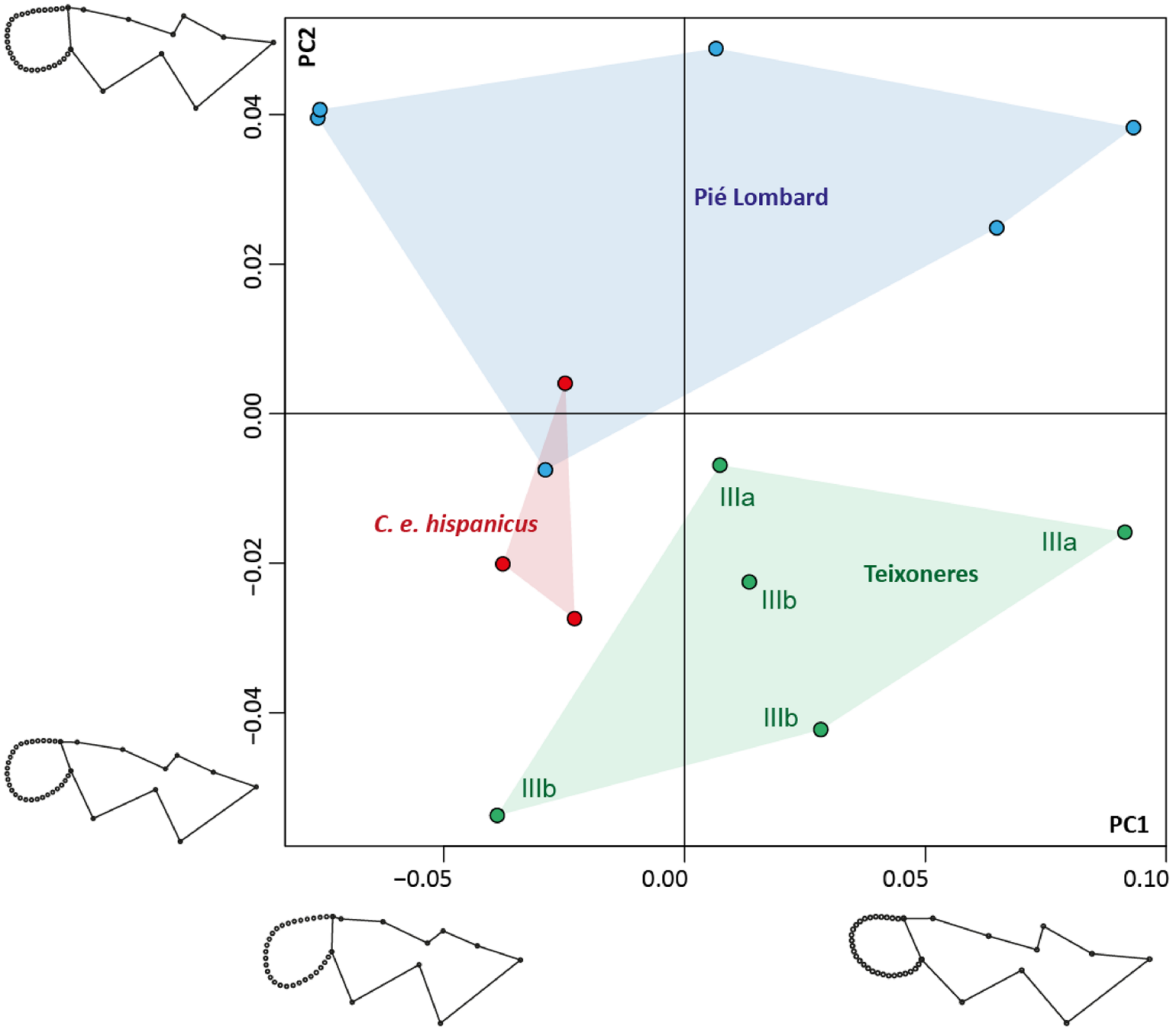
Principal components (PC) scatterplot between PC1 (48.16%) and PC2 (17.86%) performed on the shape data of the m3 according to the population (Teixoneres Unit IIIa and IIIb, Pié Lombard and current *C. e. hispanicus*) with a visualization of shape variation along PC1 and PC2.

GMM was performed on the m3 in order to envision its morphology. The main differences are displayed on the second axis of the PCA (Fig. 3). It shows the same characteristics as the m2, with these rather blunt styles on Teixoneres *Cervus* and *C. e. hispanicus* in comparison with Pié Lombard. GMM also allows us to consider the shape of the third lobe, which is difficult to evaluate qualitatively. Our analysis confirms the great morphological variability of this lobe, which can be perfectly circular or teardrop-shaped, depending on the specimen. Its position varies between the individuals from Teixoneres and those from Pié Lombard. On the latter, it seems to form a more closed angle with the second lobe, and is slightly shifted towards the lingual side. The intersection on the second and third lobes on the jugal side of Teixoneres teeth seems more open because of the elongation of the posterior style of the second lobe. Modern *C. e. hispanicus* are situated in an intermediate position between the two fossil populations on PCA axis 2.

While *Cervus* teeth display morphological differences between populations, *Capreolus* are more homogeneous. In general, the pillars of the metaconids and entoconids of the lower teeth are blunt, the parastylid rarely marked and the metastylid never expressed. The ectostylids are always present and well expressed. Cingulums are absent on the m3 but may be present on m1 and m2. Only p4 display some differences: they are always “molarised” in Layer IIIa at Teixoneres, almost always in Layer IIIb and not at Pié Lombard. Nevertheless, this character remains unreliable, as in *Cervus*^11,108^ and does not allow any real difference to be observed between the studied populations.

### Body size of *Cervus *and *Capreolus* from Pié Lombard, Teixoneres and *Cervus e. hispanicus*

The body size of the Teixoneres, Pié Lombard and *C. e. hispanicus* red deer was assessed through the study of dental and post-cranial material and compared with sub-contemporary populations from southern France and northern Spain.

The centroid size of the m3 of Teixoneres deer (Units IIIa and IIIb) and *C. e. hispanicus* is very close (Kruskal–Wallis test, *p*-value = 0.786) whereas Pié Lombard individuals have significantly larger m3 (Pié Lombard/Teixoneres *p* = 0.013; Pié Lombard/*C. e. hispanicus p* = 0.048) (Supplementary Fig. S4).

**Figure 4:**
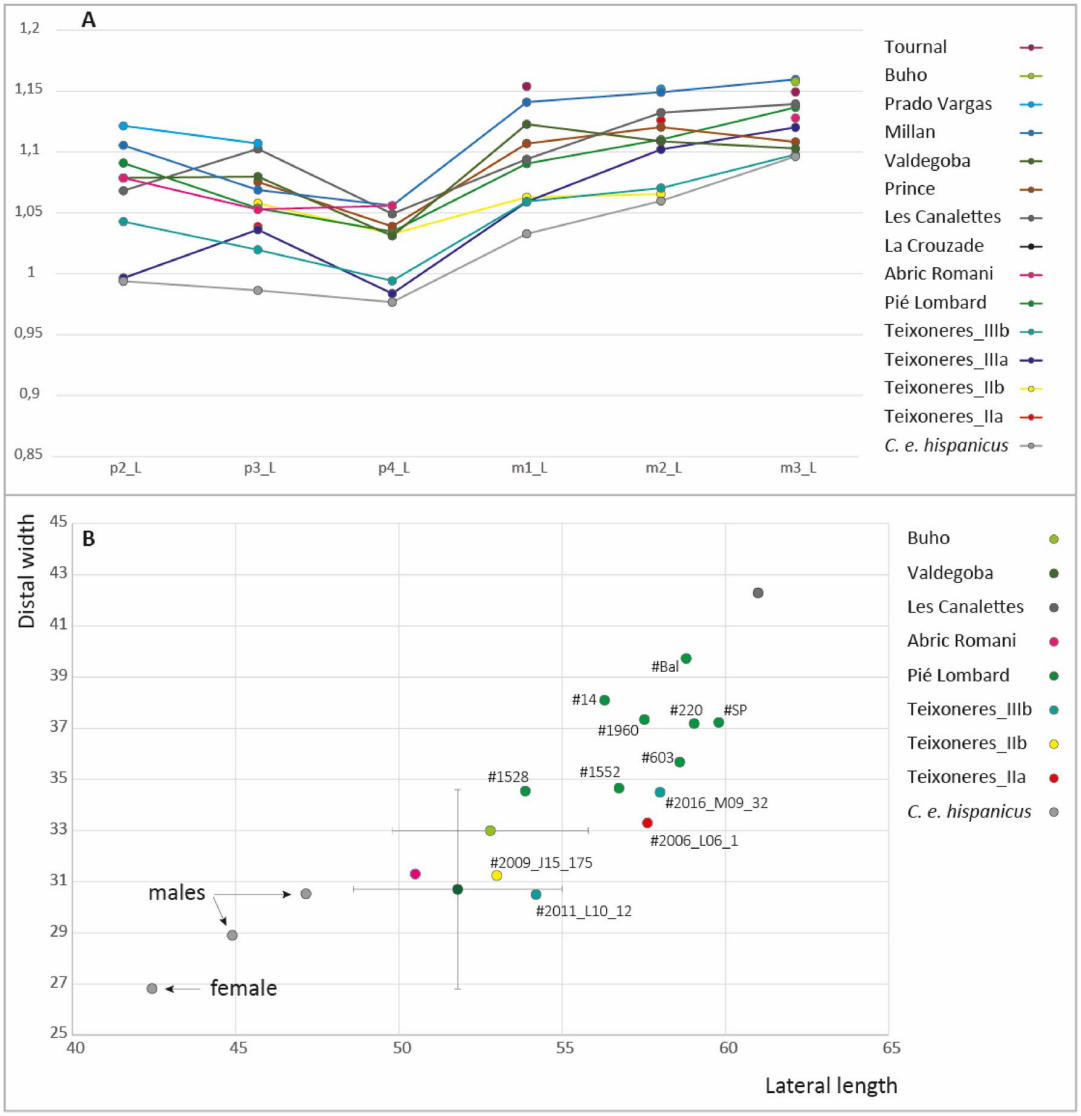
(**A**) Simpson diagram comparing the length of lower *Cervus* teeth from Teixoneres (this study), Pié Lombard (this study), *C. e. hispanicus* (this study), Grotte du Prince^112^, Abri des Canalettes^113^, Grotte de la Crouzade^114^, grotte de Tournal^74^, Cueva de Valdegoba, Level V^115^, Cueva Millan, Level 1B^115^, Cueva del Buho^115^, Prado Vargas^115^ and Abric Romani^116^. Reference 0: *Rangifer tarandus*^71^. (**B**) Scatter plot comparing the lateral length and the distal width of talus of *Cervus* from Teixoneres (this study), Pié Lombard (this study), *C. e. hispanicus* (this study), Cueva del Buho^115^, Cueva de Valdegoba, Level V^115^, Abri des Canalettes^113^ and Abric Romani^116^.

All fossil populations are larger in size than the present-day population (Fig. 4). Teixoneres deer remains particularly small, especially the individuals of Units IIIb, then IIb, IIIa and IIa. Pié Lombard individuals are smaller than the comparative fossil populations. The other Spanish populations in the south-western Pyrenees from MIS 3 are much larger, similar in size to the Canalettes (MIS 5–4, Massif Central) and the Prince's Cave (MIS 4, South West of the Alps). The biggest individual is from Tournal (MIS 3, North Pyrenees).

Post-cranial elements in red deer are more directly influenced by sexual dimorphism, which can be quite pronounced in this taxon, although its intensity varies according to habitat^16,110,111^. We displayed the raw data to consider individuals one by one in order to take this variable into account (Fig. 4). The modern individuals remain very small compared to the fossils, and a difference is visible between the female (the smallest in the sample) and the two males (Supplementary Table S7). The intra-population variability of the measurements reflects the probable presence of both males and females. The two talus from unit IIIb of Teixoneres display differences in size indicating that #2011_L10_12 would have belonged to a female and #2016_M09_32, to a male. Talus #2009_J15_175 from the unit IIb shows a size similar to #2011_L10_12 and may belong also to a female while #2006_L06_1 from the unit IIa displays larger dimensions close to #2016_M09_32 and may have belong to a male. At Pié Lombard, the variability of the talus size is greater than in Teixoneres, indicating the presence of both male and female but with no clear separation between two groups. Nevertheless, only the smaller bones (#1528 and #1552) are of comparable dimension with the ones from Teixoneres, all the other ones displaying greater sizes. Thus, despite this dimorphism, trends in the size of deer in northern Spain and southern France are apparent. The Spanish populations in the south-west and south-east of the Pyrenees are placed between the *C. e. hispanicus* and the populations north of the Pyrenees. In Teixoneres, there is a slight difference in size between the individuals in Unit IIa, which are slightly larger than those in Units IIb and IIIb in the talus. Next come the populations of Pié Lombard in the south-east of France and finally, Les Canalettes, in the south-west.

**Figure 5:**
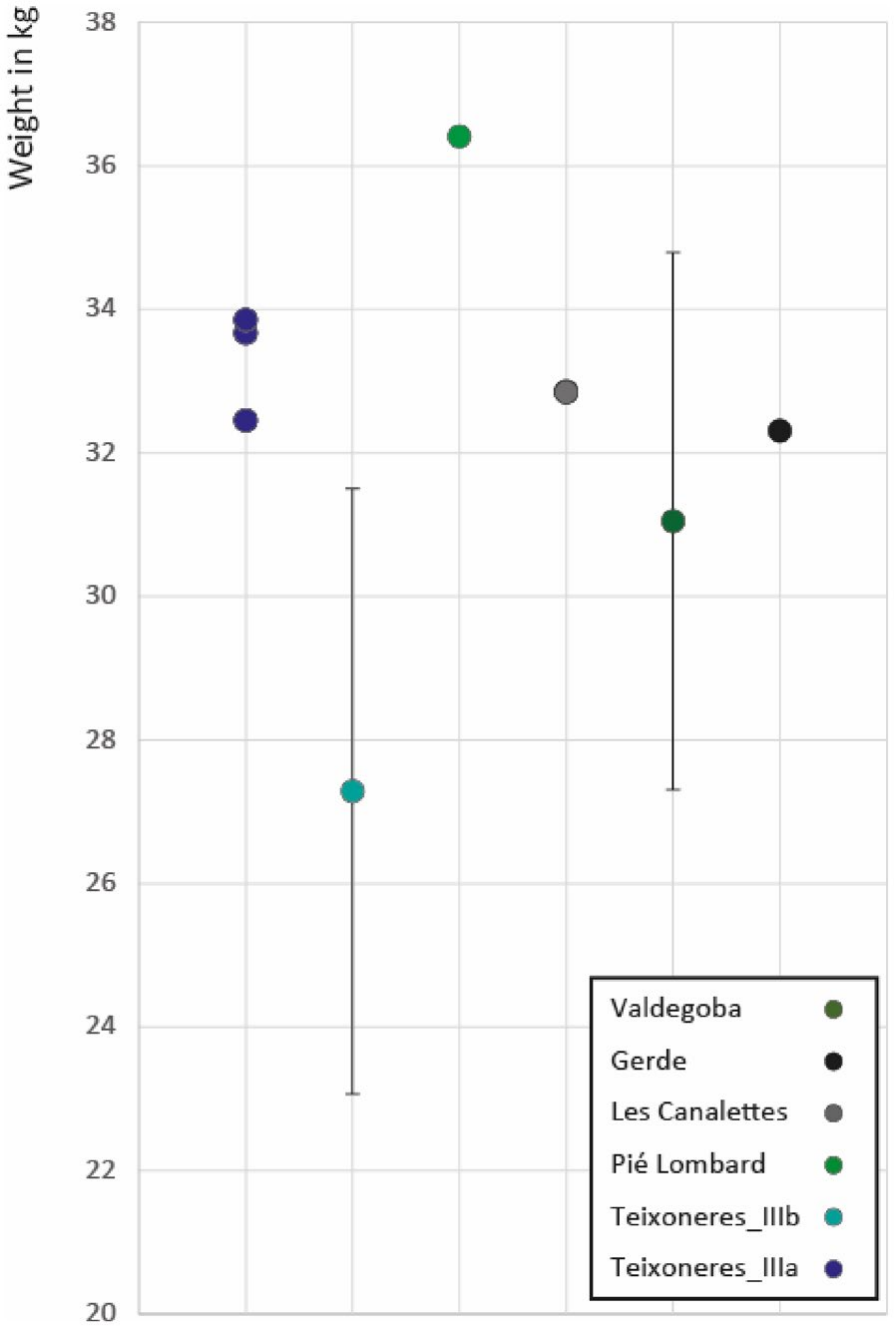
Weight estimation of *Capreolus* from Teixoneres, Pié Lombard, Cueva de Valdegoba, Level V^115^, Gerde^117^ and Abri des Canalettes^113^. Weight estimated through the measurements of M2, m1, m2 and m3 (equations from Janis^72^).

In contrast to red deer, the *Capreolus* remains are less well represented and do not allow us to fully develop the same approach. We therefore chose to estimate the body mass of individuals from different teeth (M2, m1, m2 and m3) in order to compare the series (Supplementary Table S8). We also estimate the body mass of roe deer from Canalettes, Valdegoga and Gerde, where raw data was available^113,115,117^. Sexual dimorphism in roe deer is low and can be considered negligible^23,118,119^. The body mass differences are limited and range from 27 to 36 kg. They therefore exceed their present-day counterparts, whose mass rarely exceeds 30 kg^27^. The lightest individuals come from Unit IIIb of Teixoneres and Valdegoba. The individuals from Unit IIIa of Teixoneres, those from Canalettes and Gerde, share a similar size. Finally, the individual from Pié Lombard is the heaviest (Fig. 5).

### Ecological adaptations of *Cervus *and *Capreolus* from Pié Lombard and Teixoneres

#### Cervus

The ecological adaptations of *Cervus* from Pié Lombard and Teixoneres were addressed in two ways: firstly based on the conformation of the third phalanx, and secondly based on the reconstruction of their diet. The combination of these two approaches makes it possible to address both the hardness of the substrate and their selection of plant species according to two temporal scales.

**Figure 6:**
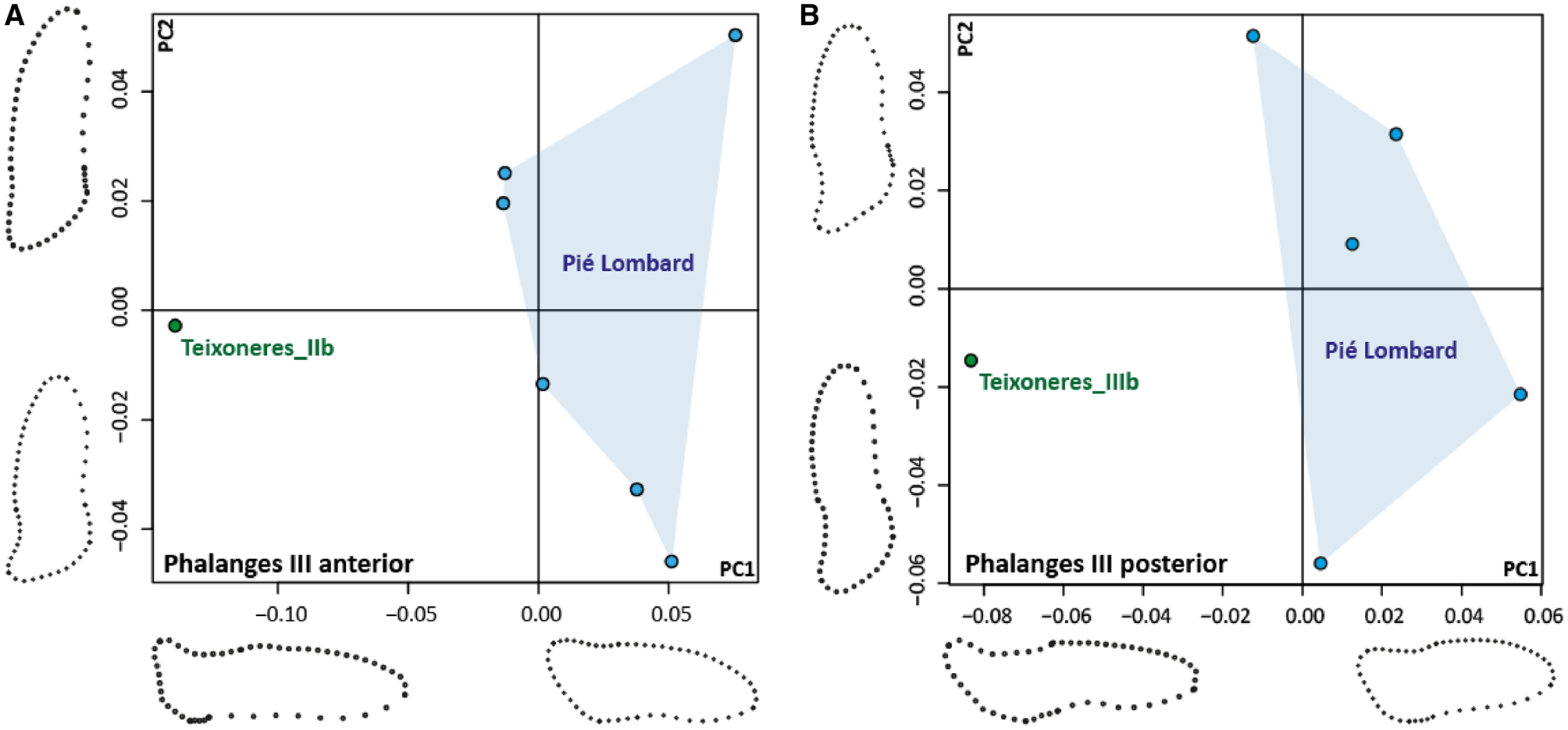
Principal components (PC) scatterplot with a visualisation of shape variation along PC1 and PC2: A: PC1 (65.38%) and PC2 (15.27%) performed on the shape data of the third anterior phalanges according to the population (Teixoneres Unit IIb and Pié Lombard). B: PC1 (50.83%) and PC2 (35.36%) performed on the shape data of the third posterior phalanges according to the population (Teixoneres Unit IIIb and Pié Lombard).

Significant differences are visible, even in a small sample, in both the anterior and posterior third phalanges of Pié Lombard and Teixoneres. Those of the Pié Lombard individuals are shorter and wider than those of the Teixoneres individuals (Units IIIb for the posterior and IIb for the anterior), which are proportionally longer and more tapered (Fig. 6).

**Figure 7:**
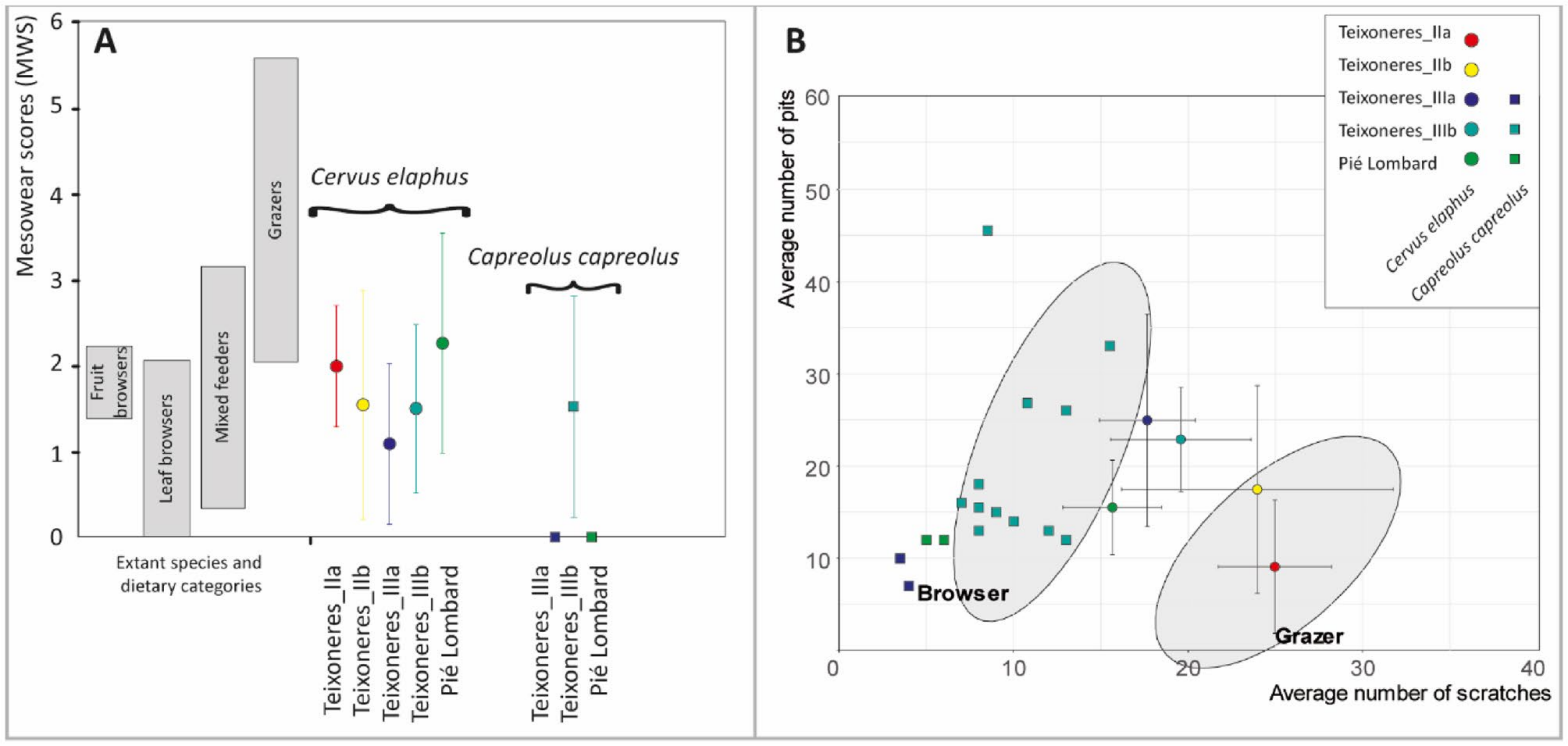
(**A**) Mesowear score (MWS) of *Cervus* and *Capreolus* from Pié Lombard and Teixoneres compared to the values of recent ungulates published by Fortelius and Solounias^62^, Solounias and Semprebon^102^, Rivals et al.^121,122^. (**B**) Bivariate plot of the mean number of pits and scratches of *Cervus* and *Capreolus* from Pié Lombard and Teixoneres. The error bars correspond to the standard deviation (± 1 SD). The ellipses correspond to the Gaussian confidence ellipse (*p *= 0.95) on the centroids of current grazers and browsers published by Solounias and Semprebon^102^.

According to the dental mesowear, the diet of red deer appears quite homogeneous (Table 1). In Teixoneres, they are all browsers with some minor variations. The most browsing individuals (MWS = 0.87) are in Unit IIIa of Teixoneres, then in Unit IIIb (MWS = 1.60), and Units IIa and IIb are represented respectively by very few teeth and have an equivalent score (MWS = 2 and 1.7 respectively). In Pié Lombard, the score (MWS = 2.70) is slightly higher and falls within the variability of mixed-feeders (Fig. 7). Nevertheless, it is still quite low and this population seems to have concentrated on low abrasion food.

**Table 1:** Summary of dental meso- and microwear data for *Cervus elaphus* and *Capreolus capreolus* from Teixoneres and Pié Lombard.

Site		Mesowear		Microwear							
		n	MWS	n	NP	NS	%LP	%G	SWS	%HC	%XS
***Cervus elaphus***											
Teixoneres_IIa	m	5	2	4	9	25	0	0	0.25	0	100
	s		0.7		7.22	3.24					
Teixoneres_IIb	m	9	1.6	9	17.38	24	22.22	0	0.33	0	77.7
	s		1.33		11.25	7.76					
Teixoneres_IIIa	m	11	1.09	14	24.96	17.64	78.57	0	0.71	7.14	64.28
	s		0.94		11.54	2.75					
Teixoneres_IIIb	m	81	1.5	62	22.87	19.57	74.19	17.74	0.92	14.52	78.69
	s		0.98		5.7	4.03					
Pié Lombard	m	63	2.27	30	15.43	15.7	16.67	23.33	0.84	3.33	70
	s		1.29		5.12	2.82					
***Capreolus capreolus***											
Teixoneres_IIIa	#2009_K16-53		0		7	4	100	0	0	0	100
	#2011_N08-36		0		10.5	6	0	0	0	0	0
Teixoneres_IIIb	m	36	1.53	12	20.66	10.25	16.67	0	0.83	0	8.33
	s		1.3		10.27	2.64					
Pié Lombard	#F3.2		0		12	6	0	0	100	0	100
	No ID		0		12	5	100	0	0	0	0

Abbreviations: # = identifier of the teeth; n = Number of specimens; MWS = Mesowear score; NP = Mean number of pits; NS = Mean number of scratches; %LP = Percentage of specimens with large pits; %G = Percentage of specimens with gouges; SWS = scratches width score; %HC = Percentage of specimens with hypercoarse scratches; %XS = Percentage of specimens with cross scratches; m = Mean; s = Standard deviation.

The variability in diet is much greater according to the dental microwear scale (Fig. 7). In Teixoneres there is a gradient where the number of pits decreases as the number of scratches increases. In Unit II, the red deer is a grazer: they have many scratches and few pits. These characteristics are particularly marked in Unit IIa. In Unit III, red deer are mixed feeders, with a medium number of scratches and a large number of pits, especially in Unit IIIa. They are, moreover, characterised by the recurrent presence of large pits (85.71 and 70.9% of the teeth) typical of the mixed-feeder diet^120^. Finally, they are also mixed-feeders at Pié Lombard, but with much less pits than in Teixoneres III. They also very often bear gouges which could indicate the ingestion of grit on a regular basis.

#### Capreolus

The roe deer at Pié Lombard and Teixoneres are always browsers, whether at meso- or micro-wear scales (Fig. 7). In Pié Lombard and Unit IIIa from Teixoneres, the index is always 0 for the two teeth available, and in Unit IIIb from Teixoneres, it is equal to 1.59 (Table 1). According to microwear analysis, the roe deer are always within the variability of browsers, with those of Pié Lombard and Unit IIIa of Teixoneres characterised by particularly few scratches and pits, while the individuals of Unit IIIb show a little more. The SWS is also higher in the individuals from IIIb.

## Discussion

Many differences in morphology, body-size and ecological adaptations have been observed among *C. elaphus* populations. Morphological and GMM analyses of red deer lower teeth have revealed qualitative and quantitative differences between Pié Lombard, Teixoneres and *C. e. hispanicus* populations. Except for the strong expression of entostyles/ectostylids in *C. e. hispanicus* when present, this subspecies is characterised by very simple teeth with very blunt styles and pillars, a mostly absent anterior fold, and a drop-shaped third lobe of the m3 that forms a closed angle with the second lobe. These characteristics contrast with Pié Lombard's *Cervus*, in which the lower teeth are much more morphologically-complex and the m3 third lobe rounder, with a more open angle with the second lobe. Red deer remains were unevenly distributed along the Teixoneres stratigraphic sequence with a majority coming from unit IIIb, however, this cohort from units IIa to IIIb appears morphologically quite homogenous. The red deer coming from all the units of Teixoneres share great proximity with the *C. e. hispanicus*, although these morphological characteristics are less systematically expressed and exacerbated. The morphometrical characteristics of *Cervus elaphus* from Teixoneres, however, differ from *C. e. hispanicus*, indicating that these two groups do not belong to the same subspecies. The morphological similarities between the two populations could indicate an ancestral relationship where the Teixoneres deer represent an archaic stage in the subspeciation of *C. e. hispanicus*.

Genetic studies place the moment of divergence between Iberian and West European deer as during the LGM^14^ or slightly earlier, during MIS 3^13^. The dating of the Teixoneres Unit IIIb ranges from ca. 45,000 BP to earlier than 51,000 BP^51^. Unit IIIb of Teixoneres could thus extend from MIS 4 to 3 covering the time frame corresponding to important intermingling of European *Cervus* populations^13^. Yet, our study points to important morphological differences between Pié Lombard and Teixoneres deer that may indicate the onset of subspeciation of *C. e.* hispanicus at least in North eastern Iberia with respect to a western European stock.

In addition to this morphological proximity, the Teixoneres red deer and *C. e. hispanicus* have a similar small body size, especially compared to the Pié Lombard individuals which are quite bigger. We estimated the relative body size of deer populations on both sides the Pyrenees from the dimensions of teeth and talus. There is not always a linear relationship between the size of the dental material and that of the post-cranial elements. The deer of the south-western Pyrenees are characterised by large teeth and small post-cranial material. Indeed, the Valdegoba deer have teeth larger than those of the Canalettes for example, but are much smaller according to their talus. This does not exist in other populations where the relative dimensions of teeth and bones are consistent with each other. We have observed slight variations in size among early Late Pleistocene red deer with an Iberian stock of small individuals (including small individuals with "big teeth" in the northwest of the peninsula), slightly larger populations in southeastern France (Pié Lombard, Prince) and even larger at the northern edge of the Pyrenees (Canalettes, Tournal). All these populations largely dominate in size the current Iberian stock.

Variations in the body-size of Pleistocene red deer have already been reported in different European regions^19,20,123^ in relation with climatic environments or the coexistence and intermingled of several haplogroups. The diversity in the body-mass of early Late Pleistocene red deer can be explained by their geographical origin, which could reflect the cohabitation of several haplogroups in Western Europe. The morphological differences between the specimens from Pié Lombard and Teixoneres support the idea that they could belong to two different genetic groups. The slightly larger size of *Cervus* in the south-western Pyrenees compared to those in the south-eastern Pyrenees may reflect this facilitated exchange of populations between the north and south of the mountain range. Moreover, genomic studies already point out the presence of a wide range of genomic lineages from all over Western Europe^15^. On the contrary, the smaller size of the south-eastern Pyrenees red deer could indicate a greater isolation of these populations.

Currently, the body size of deer varies significantly according to complex mechanisms. Population density^124,125^ or the male/female ratio^16,126^ may be key-factors. A climate that limits access to food resources due to low temperatures or drought^127–129^ may also have a negative impact on body size. In some environments, a large body size may also be favoured to allow access to advantageous food resources. For example, in Mediterranean environments, taller individuals are favoured seasonally because their size allows them to maintain a browsing diet throughout the year, whereas smaller individuals must supplement their diet with grasses^130^.

However, the observed body-size variations cannot be explained by dietary preferences. Indeed, at both Pié Lombard and Teixoneres, red deer generally concentrated on low abrasive foods. Seasonally, their diet varied from mixed feeders at Pié Lombard and Teixoneres (Unit IIIa and IIIb) to grazers at Teixoneres (Unit IIa and IIb). The higher seasonal consumption of grasses in Unit II does not seem to have particularly affected their size in comparison with Unit III where they focused on browse. Similarly, the diet of *Cervus* from Pié Lombard and Unit III of Teixoneres, as seen through tooth wear, share similar characteristics, although the specimens from Pié Lombard are larger.

Morpho-functional proxies allow to highlight a real difference in the ecological adaptations of the Pié Lombard and Teixoneres red deer. The morphology of the plantar margin of their third phalanges are distinct. The former has short, broad phalanges typical of hard terrain and open habitats and the latter have elongated, tapered phalanges of soft soil and closed environments^80,81,131^. These results are totally consistent with the environmental data from each site and might indicate that the open environment is conducive to a larger body size in red deer.

*Capreolus* is much less well represented and generally known than the red deer. Our sample nevertheless allows some preliminary observations. In contrast to *Cervus*, roe deer presents a remarkable homogeneity according to the studied parameters.

Morphologically, the two populations of Teixoneres and Pié Lombard show similar characteristics except for the p4. They are more often "molarised" in Teixoneres than in Pié Lombard, but this character is considered little diagnosis^11,108^. The proximity between the two populations does not suggest any subspeciation processes for this taxon in Southwestern Europe during MIS 4–3 that did not motivate any comparison with modern *Capreolus* material. The two populations can be attributed to *Capreolus capreolus*.

We estimated the body mass of *Capreolus* in Teixoneres, Pié Lombard and also Canalettes, Valdegoba and Gerde from published data. The lightest individuals come from layer IIIb of Teixoneres and then from Valdegoba. The individuals from layer IIIa of Teixoneres, those from Canalettes and Gerde share a similar size. Finally, the individual from Pié Lombard is the heaviest. The differences in weight are limited and range from 27 to 36 kg. This means that they are quite a bit heavier than their present-day counterparts, which rarely reach 30 kg^23^. The decrease in deer weights between the Pleistocene and Holocene has already been noted^113^ and linked to environmental factors^132,133^.

Currently, the body mass of European roe deer does not vary geographically^119,134–136^. For instance, there is no significant difference between Spanish, French, Swiss or German modern populations^27^. The variability of roe deer body mass seems to be correlated with climatic and ecological variations, the carrying capacity of the environment, the population density and the quality of their food^27,137^.

The roe deer of Pié Lombard and Teixoneres are always browsers both at the meso- or microwear scales (Supplementary Fig. S5). Although they mainly consume soft foods such as leaves, Unit IIIb *Capreolus* must have incorporated some grasses into their diet. In contrast, individuals from Pié Lombard and Teixoneres Unit IIIa may have concentrated on the exclusive consumption of leaves.

Thus, the largest individuals in our sample were able to concentrate on particularly tender dicotyledons throughout the year. The availability of these resources their capacity to focus on it may explain the development of a larger size as opposed to the smaller individuals that had to adopt a slightly more varied diet throughout the year.

*Cervus* therefore shows significant morphometric variations, whereas *Capreolus* is very stable in the early Late Pleistocene on both sides of the Pyrenees. In red deer, the differences between populations are major, heralding the separation of Iberian and West European populations and the appearance of *C. e. hispanicus*, probably towards the end of MIS 3^13^. Teixoneres specimens could express the very beginning of the subspeciation leading to *C. e. hispanicus*. The body size of these ungulates also seems to be organised geographically, perhaps reflecting the presence of different haplogroups and/or rapid body adaptations (observable on their phalanges III for example). It is not yet possible to link the variation in body size of *Cervus* to any particular environmental factor (diet or paleo-climate). *Capreolus*, on the other hand, has a homogeneous morphology and the size variations observed seem to be independent of their region of origin. Our study, which should be completed in the future, indicates that the development of the size of roe deer could reflect constant access to its ideal food, soft dicotyledons. The two cervids thus follow perfectly different eco-bio-geographical logics. The different expression of environmental constraints in these two taxa could result from the opposition between the specialised *Capreolus* which is more selective of its habitats and the greater tolerance of *Cervus* for varied ecological niches^123^, which is also seen in its ability to forage a great diversity of plants. Their plasticity may have led them to undergo greater environmental pressures of various origins in areas outside their preferred habitats, causing more rapid, important and uneasy-to-predict body adaptations.

## Conclusion

Our study focusses on late Pleistocene *Cervus* and *Capreolus* by investigating their morphometrical and ecological variations from two sites in the Mediterranean area. Methodologically, we undertook analysis combining different approaches: linear and geometric morphometry, meso- and micro-tooth wear, and the estimation of body mass. Such mixed approaches are still very limited in the study of fossil mammals, but should be further developed in the future.

There are many differences between the two populations of *Cervus elaphus*. The individuals from Teixoneres were morphometrically closer to *C. e. hispanicus* than to the individuals from Pié Lombard, indicating a possible ongoing sub-speciation in Spain. The two fossil populations also display important differences in their morpho-functional adaptations and their diet, although their body mass does not seem to be correlated to the abrasiveness of their diet. We observed a more important homogeneity among the *Capreolus*, in both their body mass and their diet. For this species, it seems that there is a direct relationship between their ability to focus on soft food all year long and their body mass. This study suggests that the ability of *Cervus* to occupy a large range of ecological niches may induce rapid body change. On another hand, the diversity of the ecological niches of *Capreolus* is more restricted to environments that favour mostly browsing. The relative homogeneity of the habitats selected by *Capreolus* may imply a relative homogeneity in their anatomical features.

Red deer eco-morpho-functional responses to environmental pressure appear to be quicker and more marked on the skeleton than roe deer ones. The strong distinction between northern and southern red deer may be an indicator of the isolation of, at least, some north-eastern Iberian populations. This separation may be linked to the Pyrenean mountains which may have greatly limited the population exchanges and acted as a strong geographical barrier.

## Supplementary Information

Supplementary Information.

## Data Availability

All data needed to evaluate the conclusions in the paper are present in the paper and/or the Supplementary Materials. All the raw data can be downloaded at https://dataverse.csuc.cat/dataset.xhtml?persistentId=doi:10.34810/data224.
