# Supplementary material for: Speciated mechanism in Quaternary cervids (Cervus and Capreolus) on both sides of the Pyrenees: a multidisciplinary approach
Source: Sci Rep. 2022 Nov 23;12:20200. doi: 10.1038/s41598-022-24684-7 (PMC9684128; doi:10.1038/s41598-022-24684-7)
Supplement: Supplementary file 1 — Supplementary Information. [file 41598_2022_24684_MOESM1_ESM.docx]

**Speciated mechanism in Quaternary Cervids (*Cervus* and *Capreolus*) on both sides of the Pyrenees: a multidisciplinary approach**

Antigone Uzunidis^1*^, Anna Rufà^2;3^, Ruth Blasco^1,4^, Jordi Rosell^1,4^, Jean-Philip Brugal^5^, Pierre-Jean Texier^5^, Florent Rivals^1,4,6^

^1^ Institut Català de Paleoecologia Humana i Evolució Social (IPHES-CERCA), Zona Educacional 4, Campus Sescelades URV (Edifici W3) 43007 Tarragona, Spain, [antigone.uzunidis@wanadoo.fr](mailto:antigone.uzunidis@wanadoo.fr)

^2^ ICArEHB – Interdisciplinary Centre for Archaeology and the Evolution of Human Behaviour, Universidade do Algarve, Campus de Gambelas 8005-139 Faro, Portugal.

^3^ Univ. Bordeaux, CNRS, MCC, PACEA, UMR 5199, F-33600 Pessac, France

^4^ Departament d’Història i Història de l’Art, Universitat Rovirai Virgili, Av. Catalunya 35, 43002 Tarragona, Spain

^5^ CNRS, Aix-Marseille Université, Minist. Cult., UMR 7269 LAMPEA, F13097 Aix-en-Provence Cedex 2

^6^ ICREA, Pg. Lluís Companys 23, 08010 Barcelona, Spain

**SUPPLEMENTARY INFORMATION – section 1**

**Topographical information about Teixoneres and Pié Lombard**


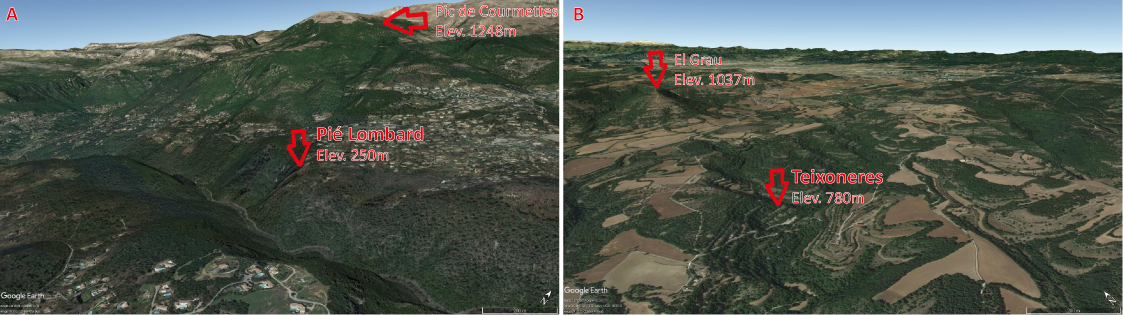


Fig. S1: Location of Pié Lombard (A) and Teixoneres (B) sites in their topographical environment. Image Data: G, 2022 CNES/Airbus

**SUPPLEMENTARY INFORMATION – section 2**

**Material**

| Site | Teixoneres_IIa | | Teixoneres_IIb | | Teixoneres_IIIa | | Teixoneres_IIIb | | Pié Lombard | |
| --- | --- | --- | --- | --- | --- | --- | --- | --- | --- | --- |
| Species | *C. elaphus* | *C. capreolus* | *C. elaphus* | *C. capreolus* | *C. elaphus* | *C. capreolus* | *C. elaphus* | *C. capreolus* | *C. elaphus* | *C. capreolus* |
| D2 |  |  |  |  | 4 |  | 13 |  | 5 |  |
| D3 | 1 |  | 1 |  | 5 |  | 22 |  | 5 |  |
| D4 | 1 |  | 2 |  | 3 |  | 14 | 3 | 6 | 1 |
| P2 | 1 |  |  |  |  |  | 9 | 6 | 6 |  |
| P3 | 1 |  |  | 1 | 1 | 1 | 15 | 4 | 5 |  |
| P4 |  |  | 3 |  | 5 | 1 | 16 | 8 | 8 |  |
| M1 | 2 |  | 1 |  | 1 |  | 12 | 5 | 6 |  |
| M2 |  |  | 2 |  | 3 |  | 22 | 5 | 5 |  |
| M3 | 1 |  | 2 |  | 6 |  | 10 | 2 | 11 |  |
| d2 |  |  | 1 |  | 1 |  | 20 | 1 | 5 |  |
| d3 |  |  | 1 |  | 2 |  | 30 | 2 | 3 |  |
| d4 |  |  | 1 | 1 | 3 |  | 32 | 2 | 2 |  |
| p2 |  |  |  |  | 2 |  | 12 | 2 | 14 |  |
| p3 | 1 |  | 1 |  |  |  | 12 | 7 | 12 | 1 |
| p4 |  |  | 1 |  | 3 | 1 | 21 | 4 | 12 | 1 |
| m1 |  |  | 1 |  | 2 | 1 | 7 | 3 | 18 |  |
| m2 | 1 |  | 2 |  | 1 |  | 11 | 5 | 6 |  |
| m3 |  |  |  |  | 1 | 2 | 12 | 4 | 9 | 1 |
| Scapula |  |  |  |  |  |  |  |  | 4 |  |
| Humerus | 1 |  |  | 1 | 1 |  | 1 | 2 | 9 | 1 |
| Radius |  |  |  |  |  |  | 1 | 3 | 13 |  |
| Triquetrum |  |  | 1 |  |  |  |  |  | 16 |  |
| Hamatum | 1 |  |  |  |  |  |  |  | 12 |  |
| Capitatotrapezoid | 2 |  |  |  |  |  |  |  | 8 |  |
| Lunatum |  |  |  |  | 1 |  | 2 | 1 | 10 |  |
| Scaphoid |  |  |  |  | 1 |  | 2 |  | 9 |  |
| Metacarpal |  |  |  |  | 1 |  | 5 | 1 | 4 |  |
| PhI anterior | 1 |  | 1 |  |  |  | 6 |  | 8 |  |
| PhIII anterior |  |  | 1 |  |  | 1 |  | 1 | 13 |  |
| Femur |  |  | 1 | 1 |  |  | 2 | 1 | 3 | 1 |
| Tibia |  |  |  |  | 2 |  | 1 |  | 7 |  |
| Calcaneus |  |  |  |  |  |  | 1 |  | 2 |  |
| Talus | 1 |  | 2 |  |  | 1 | 2 | 3 | 13 |  |
| First cuneiform |  |  |  |  | 1 |  | 3 |  | 3 |  |
| Cubonavicular |  |  |  |  |  |  |  |  | 10 |  |
| Metatarsal |  |  |  |  |  |  | 2 |  | 3 |  |
| PhI posterior |  |  |  |  | 1 |  | 6 |  | 5 |  |
| PhIII posterior |  |  |  | 1 |  |  | 1 |  | 7 |  |
| Ph I |  |  |  |  |  |  |  | 1 |  |  |
| PhII |  |  | 1 |  | 1 |  | 6 | 3 | 26 |  |
| PhIII |  |  |  |  |  |  |  | 1 |  |  |

Table S1: Anatomical representation of dental and bone elements of *Cervus* and *Capreolus* in Pié Lombard (Ensemble II) and Teixoneres (Units IIb, IIIa and IIIb).

| **Site** | **Location/region** | **Chronology** | **Species** | **References** |
| --- | --- | --- | --- | --- |
| Grotte du Prince | South-East France | MIS 4 | *Cervus elaphus* | Alonso-Moullé, 1997-1998 |
| Abri des Canalettes | North Pyrenees | MIS 5-4 | *Cervus elaphus* | Brugal, 1993 |
|  |  |  | *Capreolus capreolus* |  |
| Gerde | North Pyrenees | MIS 4 | *Capreolus capreolus* | Clot, 1989 |
| Grotte de la Crouzade | North Pyrenees | MIS 4-3 | *Cervus elaphus* | Gerber, 1972 |
| Grotte de Tournal | North Pyrenees | MIS 3 | *Cervus elaphus* | Magniez, 2010 |
| Cueva de Valdegoba, level V | South-West Pyrenees | MIS 3 | *Cervus elaphus* | Alonso, 2005 |
|  |  |  | *Capreolus capreolus* |  |
| Cueva Millan, level 1B | South-West Pyrenees | MIS 3 | *Cervus elaphus* | Alonso, 2005 |
| Cueva del Buho | South-West Pyrenees | MIS 3 | *Cervus elaphus* | Alonso, 2005 |
| Prado Vargas | South-West Pyrenees | MIS 3 | *Cervus elaphus* | Alonso, 2005 |
| Abric Romani | South-East Pyrenees | MIS 3 | *Cervus elaphus* | Sanchez, 1989 |
| IPHES osteological collection | Spain | current | *Cervus elaphus hispanicus* | This work |

Table S2: Summary of the comparison populations used in this work with their geographical positions, dates, species involved.

**SUPPLEMENTARY INFORMATION – section 2**

**Method**

| **Species** | **Element** | **Type of analysis** | **Teixoneres  Unit IIa** | **Teixoneres Unit IIb** | **Teixoneres Unit IIIa** | **Teixoneres Unit IIIb** | **Pié Lombard** | ***C. e. hispanicus*** |
| --- | --- | --- | --- | --- | --- | --- | --- | --- |
| *C. elaphus* | upper P2 | Morphology | 1 |  |  | 9 | 6 | 7 |
|  | upper P3 | Morphology | 1 |  | 1 | 15 | 5 | 8 |
|  | upper P4 | Morphology |  | 3 | 5 | 16 | 8 | 8 |
|  | upper M1 | Morphology | 2 | 1 | 1 | 12 | 5 | 10 |
|  | upper M2 | Morphology |  | 2 | 3 | 22 | 5 | 10 |
|  | upper M3 | Morphology | 1 | 2 | 6 | 10 | 11 | 8 |
|  | lower p2 | Linear morphometry |  |  | 2 | 12 | 14 | 7 |
|  | lower p3 | Linear morphometry | 1 | 1 |  | 12 | 12 | 6 |
|  | lower p4 | Linear morphometry |  | 1 | 3 | 21 | 12 | 7 |
|  | lower m1 | Linear morphometry |  | 1 | 2 | 7 | 18 | 10 |
|  | lower m2 | Linear morphometry,  Morphology | 1 | 2 | 1 | 11 | 6 | 10 |
|  | lower m3 | Linear morphometry,  GMM |  |  | 1 | 12 | 9 | 7 |
|  | talus | Linear morphometry | 1 | 1 |  | 2 | 13 | 3 |
|  | third phalanx | GMM |  | 1 |  | 1 | 11 |  |
|  | Upper and lower teeth | Dental mesowear | 5 | 9 | 11 | 81 | 63 |  |
|  | Upper and lower teeth | Dental microwear | 4 | 9 | 14 | 62 | 30 |  |
| *C. capreolus* | upper P2 | Morphology |  |  |  | 6 | 1 |  |
|  | upper P3 | Morphology |  | 1 | 1 | 4 |  |  |
|  | upper P4 | Morphology |  |  | 1 | 6 |  |  |
|  | upper M1 | Morphology |  |  |  | 3 |  |  |
|  | upper M2 | Morphology,  weight estimation |  |  |  | 5 |  |  |
|  | upper M3 | Morphology |  |  |  | 2 |  |  |
|  | lower p2 | Morphology |  |  |  | 2 |  |  |
|  | lower p3 | Morphology |  |  |  | 7 | 1 |  |
|  | lower p4 | Morphology |  |  | 1 | 3 | 1 |  |
|  | lower m1 | Morphology,  weight estimation |  |  | 1 | 3 |  |  |
|  | lower m2 | Morphology,  weight estimation |  |  |  | 5 |  |  |
|  | lower m3 | Morphology,  weight estimation |  |  | 1 | 4 | 1 |  |
|  | Upper and lower teeth | Dental mesowear |  |  | 2 | 39 | 2 |  |
|  | Upper and lower teeth | Dental microwear |  |  | 2 | 12 | 2 |  |

Table S3: Summary of the teeth and bone elements used in this study with their number and the type of analysis applied to them.

**
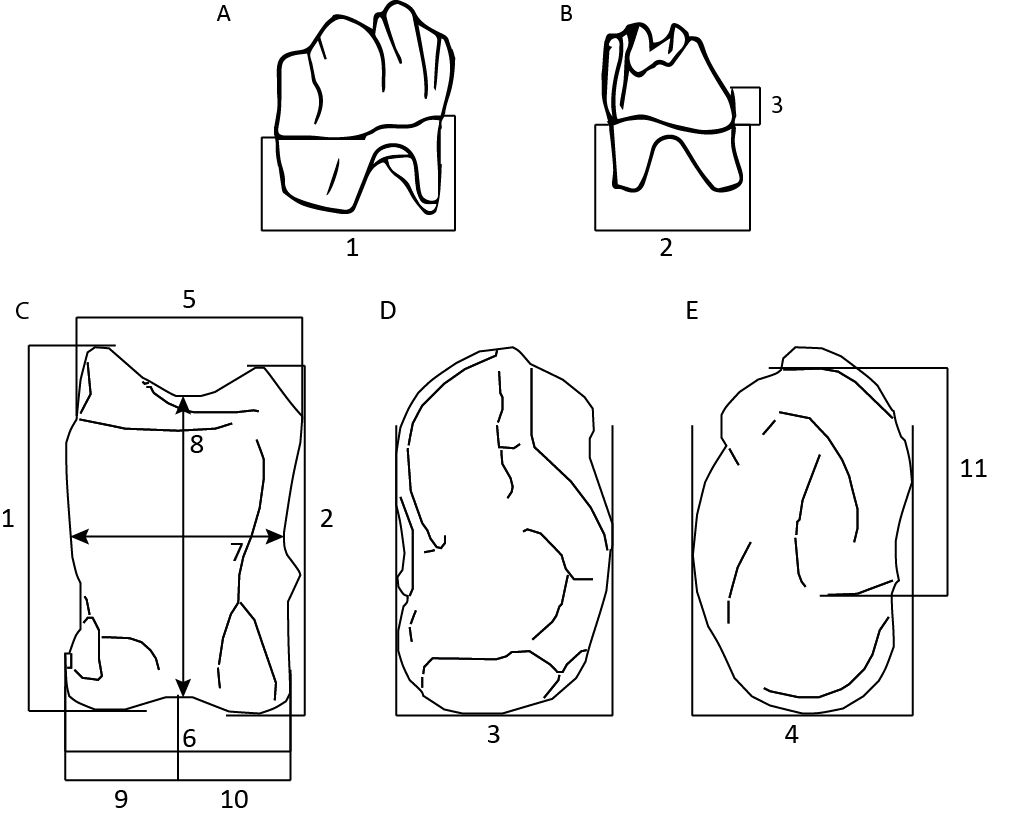
**

Fig. S2: Figurations of the measurements taken on cervid teeth (A and B) and talus (C, D and E). A-1: greatest length at the collar; B-2: greatest width at the collar; B-3: height of the entostyle/ectostylid; C-1: lateral length; C-2: medial length; D-3: dorso-plantar lateral thickness; E-4: dorso-plantar medial thickness; C-5: proximal width; C-6: distal width; C-7: middle width; C-8: middle length; C-9: lateral distal trochlea length; C-10: medial distal trochlea length; E-11: medio-proximal articular facet length.

**
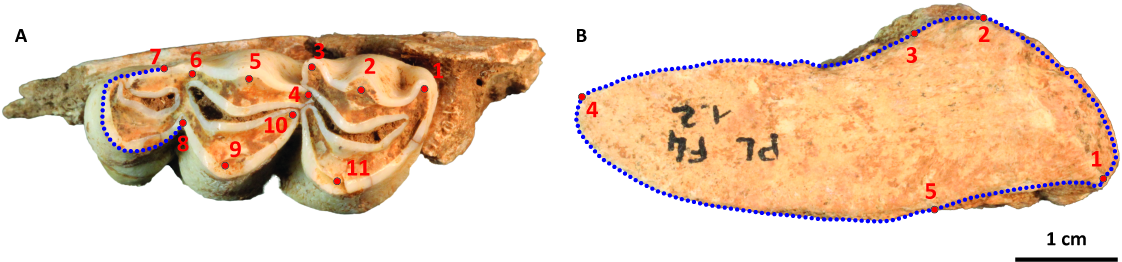
**

Fig. S3: Location of anatomical landmarks (red spheres), curve sliding (blue spheres) semilandmarks placed on the m3 (A) and third phalanx (B). A: All the landmarks are placed on the inner part of the enamel except for the semilandmarks and landmarks 7 and 8 which are on the outside part of the enamel. A-1: maximum curvature of the parastylid; A-2: maximum curvature of the metaconid; A-3: maximum curvature of the metastylid; A-4: maximum curvature of the pre-entocristid; A-5: maximum curvature of the entoconid; A-6: maximum curvature of the post-entocristid; A-7: maximum constriction before the entononulid on the jugal side; A-8: maximum constriction before the entononulid on the lingual side; A-9: maximum curvature of the protoconid; A-10: junction point between the first two lobes; A-11: maximum curvature of the hypoconid. B-1: junction point between the postero-plantar crest and the most postero-medial extension of the plantar margin; B-2: junction point between the postero-plantar crest and the most postero-lateral extension of the plantar margin; B-3: point before the medial curvature; B-4: most distal extension on the medial side; B-5: point before the lateral curvature.

**SUPPLEMENTARY INFORMATION – section 3**

**Results: Morphological characteristics of *Cervus* and *Capreolus* from Pié Lombard, Teixoneres and *Cervus e. hispanicus* teeth**

| **Site** |  | **P2_L** | **P2_w** | **P3_L** | **P3_w** | **P4_L** | **P4_w** | **M1_L** | **M1_w** | **E_H** | **M2_L** | **M2_w** | **E_H** | **M3_L** | **M3_w** | **E_H** |
| --- | --- | --- | --- | --- | --- | --- | --- | --- | --- | --- | --- | --- | --- | --- | --- | --- |
| Teixoneres |  | 15,9* | 14,95* | 15,62* | 17,21* |  |  | 21* | 24,25* |  |  |  |  | 21,5* |  |  |
| IIa |  |  |  |  |  |  |  | 19,52* | 23,24* |  |  |  |  |  |  |  |
| Teixoneres |  |  |  |  |  | 15,07* | 19,36* | 16,46* | 16,8* | 1,76* | 20,58* | 22,82* | 6,91* | 23,5* | 25,54* |  |
| IIb |  |  |  |  |  | 14,15* | 17,5* |  |  |  | 23,86* | 25,34* | 7* | 21,96* | 24,07* |  |
|  |  |  |  |  |  | 14,64* | 19,74* |  |  |  |  |  |  |  |  |  |
| Teixoneres | n |  |  |  | 16,11* | *4* | *5* | 19,93* |  | 4,72* | *3* | *2* | *1* | *6* | *6* | *5* |
| IIIa | m |  |  |  |  | **14,69** | **17,40** |  |  |  | **21,82** | **23,57** | **7,95** | **23,22** | **22,01** | **5,98** |
|  | s |  |  |  |  | 1,16 | 2,76 |  |  |  | 1,60 | 2,24 |  | 3,65 | 2,45 | 1,87 |
|  | min |  |  |  |  | 13,60 | 14,21 |  |  |  | 20,00 | 21,98 |  | 19,74 | 18,15 | 3,70 |
|  | max |  |  |  |  | 16,32 | 19,75 |  |  |  | 23,02 | 25,15 |  | 27,64 | 24,62 | 8,90 |
| Teixoneres | n | *8* | *9* | *15* | *15* | *14* | *16* | *12* | *11* | *5* | *22* | *19* | *7* | *10* | *10* | *6* |
| IIIb | m | **15,21** | **13,87** | **14,24** | **15,21** | **14,09** | **16,12** | **19,69** | **22,34** | **4,74** | **21,66** | **22,82** | **4,83** | **21,56** | **23,35** | **6,95** |
|  | s | 1,02 | 1,21 | 1,58 | 2,02 | 1,12 | 1,87 | 1,14 | 1,23 | 2,35 | 1,64 | 1,98 | 1,55 | 1,19 | 1,49 | 2,13 |
|  | min | 13,32 | 12,34 | 12,11 | 12,52 | 12,34 | 11,90 | 17,80 | 20,76 | 2,31 | 18,47 | 19,04 | 2,69 | 19,58 | 21,10 | 4,65 |
|  | max | 16,60 | 16,73 | 16,95 | 18,85 | 15,95 | 18,83 | 21,44 | 24,41 | 8,32 | 24,20 | 25,68 | 6,74 | 23,69 | 25,86 | 9,76 |
| Pié Lombard | n | *5* | *6* | *5* | *5* | *8* | *7* | *5* | *5* | *4* | *5* | *4* | *3* | *11* | *10* | *9* |
|  | m | **16,02** | **16,34** | **16,52** | **18,19** | **15,07** | **19,29** | **20,40** | **22,68** | **3,90** | **23,28** | **24,28** | **4,47** | **24,78** | **24,95** | **7,47** |
|  | s | 1,34 | 2,10 | 1,14 | 1,92 | 1,13 | 1,41 | 0,38 | 1,49 | 1,30 | 0,66 | 0,21 | 0,90 | 1,84 | 1,66 | 2,60 |
|  | min | 14,80 | 12,70 | 15,50 | 16,00 | 13,45 | 17,32 | 20,10 | 21,00 | 2,50 | 22,50 | 24,10 | 3,60 | 22,90 | 21,70 | 4,45 |
|  | max | 18,00 | 18,60 | 17,90 | 21,10 | 17,20 | 21,00 | 21,00 | 25,00 | 5,34 | 24,30 | 24,50 | 5,40 | 27,90 | 27,85 | 11,70 |
| *C. e. hispanicus* | n | *7* | *7* | *8* | *8* | *8* | *8* | *10* | *10* | *5* | *10* | *10* | *4* | *8* | *8* | *6* |
|  | m | **14,77** | **12,99** | **13,87** | **15,37** | **12,67** | **17,22** | **17,12** | **19,26** | **4,97** | **20,46** | **20,45** | **5,38** | **20,68** | **20,46** | **6,50** |
|  | s | 0,99 | 0,94 | 0,43 | 1,07 | 0,91 | 1,54 | 1,53 | 1,16 | 1,07 | 1,57 | 1,11 | 3,21 | 0,97 | 1,35 | 2,69 |
|  | min | 13,81 | 12,00 | 13,40 | 13,70 | 11,50 | 15,60 | 15,54 | 17,11 | 3,87 | 18,20 | 18,36 | 0,62 | 19,33 | 18,19 | 3,20 |
|  | max | 16,35 | 14,78 | 14,50 | 16,50 | 14,25 | 19,67 | 19,60 | 20,87 | 6,16 | 22,41 | 21,80 | 7,60 | 21,94 | 22,02 | 9,70 |

Table S4: Summary of the measurements of the upper permanent teeth of *C. e. hispanicus* and *Cervus* from Teixoneres and Pié Lombard. L = length, w = width, E_H = height of the entostyle. n = sample size; m = mean; s = standard deviation; min = minimum; max = maximum. Measurements with an asterisk are raw data. All the measurements are taken at the base of the crown.

| **Site** |  | **P2_L** | **P2_w** | **P3_L** | **P3_w** | **P4_L** | **P4_w** | **M1_L** | **M1_w** | **E_H** | **M2_L** | **M2_w** | **E_H** | **M3_L** | **M3_w** | **E_H** |
| --- | --- | --- | --- | --- | --- | --- | --- | --- | --- | --- | --- | --- | --- | --- | --- | --- |
| Teixoneres |  |  |  | 15.61* | 9.98* |  |  |  |  |  | 25* | 14* |  |  |  |  |
| IIa |  |  |  |  |  |  |  |  |  |  |  |  |  |  |  |  |
| Teixoneres |  |  |  | 16.42* | 10.64* | 18.08* | 11.6* | 19.54* | 11.84* | 2.5* |  | 13.67* |  |  |  |  |
| IIb |  |  |  |  |  |  |  |  |  |  | 21.03* | 13.2* |  |  |  |  |
| Teixoneres | n | 10,22* | 6,5* |  |  | *3* | *3* | 19,32* | 12,4* |  | 24,8* | 15,3* |  | 34,03* | 14,28* |  |
| IIIa | m | 10,25* | 5,74* |  |  | **14,91** | **9,67** |  | 12,36* | 1,56* |  |  |  |  |  |  |
|  | s |  |  |  |  | 1,07 | 1,31 |  |  |  |  |  |  |  |  |  |
|  | min |  |  |  |  | 14,03 | 8,40 |  |  |  |  |  |  |  |  |  |
|  | max |  |  |  |  | 16,11 | 11,01 |  |  |  |  |  |  |  |  |  |
| Teixoneres | n | *12* | *12* | *11* | *12* | *16* | *21* | *6* | *7* |  | *8* | *11* | *1* | *11* | *12* | *4* |
| IIIb | m | **11,41** | **7,14** | **14,70** | **8,75** | **16,37** | **10,15** | **19,35** | **12,57** |  | **20,83** | **13,58** | **3,20** | **29,60** | **13,56** | **4,26** |
|  | s | 1,13 | 0,80 | 1,40 | 1,05 | 1,46 | 1,21 | 1,59 | 0,48 |  | 1,74 | 1,89 |  | 1,74 | 2,07 | 1,06 |
|  | min | 9,47 | 5,79 | 11,95 | 6,61 | 14,08 | 7,28 | 17,91 | 12,09 |  | 16,83 | 8,54 |  | 25,60 | 8,00 | 3,16 |
|  | max | 13,60 | 8,52 | 16,70 | 10,06 | 18,33 | 11,70 | 22,42 | 13,52 |  | 22,41 | 15,88 |  | 31,63 | 16,70 | 5,20 |
| Pié Lombard | n | *14* | *14* | *11* | *12* | *12* | *12* | *11* | *18* | *11* | *6* | *6* | *5* | *9* | *9* | *8* |
|  | m | **12.76** | **7.21** | **16.24** | **9.90** | **18.18** | **11.94** | **21.11** | **14.09** | **4.28** | **23.90** | **15.83** | **4.20** | **33.17** | **15.20** | **3.41** |
|  | s | 1.14 | 1.08 | 0.68 | 0.70 | 1.12 | 0.68 | 1.84 | 0.87 | 1.15 | 1.42 | 0.85 | 0.59 | 2.24 | 1.24 | 1.11 |
|  | min | 11.12 | 5.60 | 15.10 | 9.00 | 16.50 | 11.00 | 18.94 | 12.80 | 2.45 | 21.60 | 14.70 | 3.57 | 30.40 | 13.70 | 2.50 |
|  | max | 14.55 | 9.00 | 17.00 | 11.00 | 20.30 | 13.00 | 23.84 | 15.50 | 6.37 | 25.15 | 17.15 | 4.85 | 36.60 | 17.70 | 6.00 |
| *C. e. hispanicus* | n | *7* | *7* | *6* | *6* | *7* | *7* | *10* | *10* | *8* | *10* | *10* | *5* | *7* | *7* | *4* |
|  | m | **10.17** | **5.37** | **13.59** | **8.62** | **15.46** | **9.88** | **17.96** | **11.32** | **3.01** | **20.69** | **12.34** | **3.32** | **29.30** | **12.75** | **3.70** |
|  | s | 0.90 | 0.37 | 0.67 | 0.50 | 1.06 | 0.30 | 1.86 | 0.76 | 1.10 | 1.77 | 0.76 | 0.95 | 2.03 | 0.59 | 1.44 |
|  | min | 8.57 | 5.00 | 12.86 | 8.20 | 14.50 | 9.40 | 15.44 | 9.80 | 1.96 | 17.60 | 11.00 | 2.17 | 25.70 | 12.17 | 2.40 |
|  | max | 11.30 | 6.11 | 14.68 | 9.47 | 17.18 | 10.30 | 20.64 | 12.13 | 4.76 | 24.17 | 13.35 | 4.37 | 31.84 | 13.90 | 5.43 |

Tabl S5: Summary of the measurements of the lower permanent teeth of *C. e. hispanicus* and *Cervus* from Teixoneres and Pié Lombard. L = length, w = width, E_H = height of the ectostylid. n = sample size; m = mean; s = standard deviation; min = minimum; max = maximum. Measurements with an asterisk are raw data. All the measurements are taken at the base of the crown.

| **Specimen** | **Projection of the metaconid pillar** | **Projection of the entoconid pillar** | **Projection of the parastylid** | **Projection of the metastylid** | **Projection of the entostylid** | **Presence of the ectostylid** | **Presence of the anterior fold** | **Presence of a cingulum** |
| --- | --- | --- | --- | --- | --- | --- | --- | --- |
| Tx_IIIb_663 | 0 | 0 | 1 | 1 | 0 | 0 | 1 | 1 |
| Tx_IIIb_391 | 0 | 0 | 0 | 1 | 0 | 1 | 1 | 1 |
| Tx_IIIb_70 | 0 | 0 | 0 | 0 | 0 | 1 | 1 | 1 |
| Tx_IIIb_336 | 0 | 0 | 0 | 0 | 0 | 1 | 1 | 1 |
| Tx_IIa_5 | 0 | 0 | 1 | 1 | 1 | 0 | 1 | 1 |
| Tx_IIIb_41 | 0 | 0 | 1 | 1 | 0 | 0 | 0 | 1 |
| Tx_IIb_227 | 0 | 0 | 0 | 0 | 0 | 1 | 1 | 1 |
| Tx_IIIb_151 | 0 | 0 | 1 | 0 | 1 | 0 | 1 | 1 |
| Tx_IIIa_55 | 0 | 0 | 1 | 0 | 0 | 0 | 1 | 0 |
| Tx_IIIb_167 | 0 | 0 | 1 | 0 | 0 | 0 | 1 | 1 |
| Tx_IIb_124 | 0 | 1 | 1 | 0 | 1 | 0 | 1 | 1 |
| Tx_IIIb_10 | 0 | 0 | 1 | 0 | 0 | 0 | 1 | 1 |
| Tx_IIIb_115 | 0 | 0 | 0 | 0 | 0 | 0 | 0 | 0 |
| Tx_IIIb_37 | 0 | 0 | 0 | 1 | 0 | 0 | 0 | 0 |
| Tx_IIIb_372 | 0 | 0 | 1 | 0 | 0 | 1 | 1 | 0 |
| IPHES_1 | 0 | 0 | 1 | 0 | 0 | 1 | 0 | 0 |
| IPHES_2 | 0 | 0 | 0 | 0 | 0 | 0 | 1 | 0 |
| IPHES_3 | 0 | 0 | 0 | 0 | 0 | 0 | 1 | 0 |
| IPHES_4 | 0 | 0 | 0 | 0 | 0 | 0 | 1 | 0 |
| IPHES_5 | 0 | 0 | 0 | 0 | 0 | 0 | 1 | 0 |
| IPHES_6 | 0 | 0 | 0 | 0 | 0 | 0 | 1 | 0 |
| IPHES_7 | 0 | 0 | 0 | 0 | 0 | 0 | 1 | 0 |
| IPHES_8 | 0 | 0 | 0 | 0 | 0 | 0 | 0 | 0 |
| IPHES_9 | 0 | 0 | 0 | 0 | 0 | 0 | 1 | 0 |
| IPHES_10 | 0 | 0 | 0 | 0 | 0 | 0 | 1 | 0 |
| PL_278 | 1 | 1 | 1 | 1 | 0 | 1 | 1 | 1 |
| PL_750 | 1 | 1 | 1 | 1 | 1 | 1 | 1 | 0 |
| PL_704 | 1 | 1 | 1 | 1 | 1 | 1 | 1 | 0 |
| PL_506 | 0 | 0 | 0 | 0 | 0 |  |  |  |
| PL_245 | 1 | 1 | 1 | 1 | 1 | 1 | 1 | 0 |
| PL_309 | 1 | 1 | 1 | 1 | 1 | 1 | 1 | 0 |

Table S6: Morphological parameters observed on the m2 of *C. e. hispanicus* and *Cervus* from Teixoneres and Pié Lombard.

**Results: Morphological characteristics of *Cervus* and *Capreolus* from Pié Lombard, Teixoneres and *Cervus e. hispanicus* teeth**

**
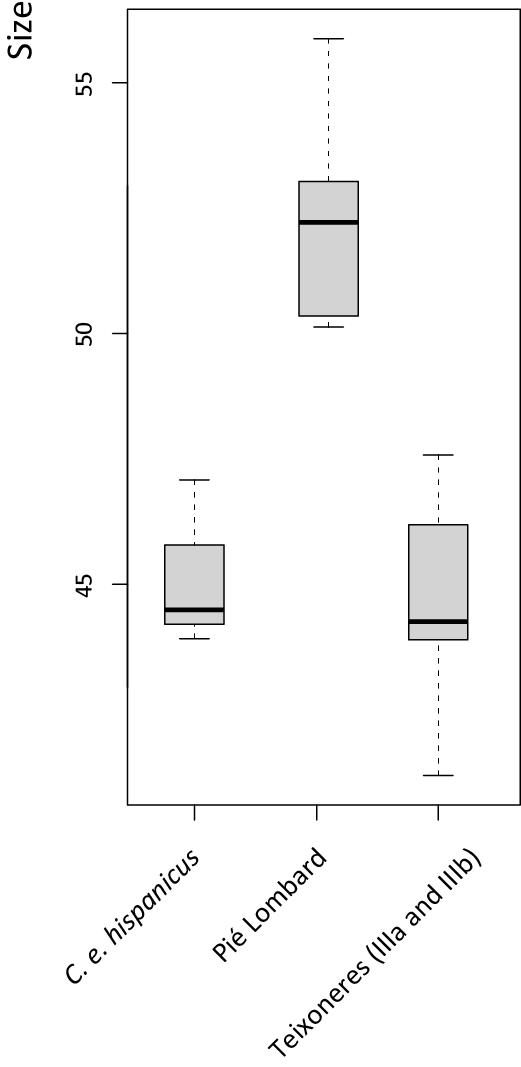
**

Fig. S4: Box plots of size variations (centroid size) of m3 of *Cervus* from Teixoneres and Pié Lombard and *C. e. hispanicus*.

**Results: Morphological characteristics of *Cervus* and *Capreolus* from Pié Lombard, Teixoneres and *Cervus e. hispanicus* teeth**

| **Site** |  | **C-1** | **C-2** | **D-3** | **E-4** | **C-5** | **C-6** | **C-7** | **C-8** | **C-9** | **C-10** | **E-11** |
| --- | --- | --- | --- | --- | --- | --- | --- | --- | --- | --- | --- | --- |
| Teixoneres_IIa |  | 57.61* | 51.7* | 30.75* |  | 32.75* | 33.3* |  | 44.5* | 17.2* | 12.5* | 31.15* |
| Teixoneres_IIb |  | 53* | 49.85* | 29.88* |  | 31.4* | 31.24* | 29.28* | 42.61* | 12.76* | 16.5* | 35.16* |
|  |  |  |  |  | 26.06* | 29.9* |  | 25.67* |  |  |  | 24.51* |
| Teixoneres_IIIb |  | 58* | 54.37* | 31.05* | 33.26* | 40.17* | 34.5* | 33.64* | 47.41* | 16.4* | 19.1* | 36.71* |
|  |  | 54.2* | 51.96* | 29.76* | 29.32* |  | 30.5* | 30.75* | 45.13* |  | 15.49* | 30.32* |
| Pié Lombard | n | *8* | *10* | *8* | *9* | *10* | *11* | *10* | *9* | *13* | *11* | *9* |
|  | m | **57.58** | **53.19** | **31.27** | **32.15** | **36.73** | **36.69** | **34.81** | **46.30** | **15.90** | **14.75** | **34.85** |
|  | s | 1.92 | 1.55 | 0.83 | 1.40 | 1.35 | 1.63 | 1.52 | 1.81 | 2.03 | 2.02 | 2.50 |
|  | min | 53.87 | 50.51 | 30.05 | 29.90 | 35.37 | 34.55 | 32.40 | 43.83 | 11.75 | 12.30 | 30.94 |
|  | max | 59.80 | 55.10 | 32.95 | 34.66 | 39.15 | 39.73 | 37.82 | 48.91 | 18.23 | 18.65 | 37.81 |
| *C. e. hispanicus* | female | 42.44* | 40.93* | 22.42* | 23.78* | 25.96* | 26.82* | 23.57* | 34.23* | 11.18* | 12* | 25* |
|  | male | 44.9* | 42.66* | 24.84* | 26.34* | 27.86* | 28.9* | 26.33* | 36.59* | 9.91* | 15.45* | 28.57* |
|  | male | 47.15* | 44.82* | 25.95* | 27.91* | 31* | 30.53* | 27.47* | 37.92* | 12.52* | 14.28* | 28.92* |

Table S7: Summary of the measurements of the talus of *C. e. hispanicus* and the *Cervus* from Teixoneres and Pié Lombard. The references for the measurements are in Figure S4. n = sample size; m = mean; s = standard deviation; min = minimum; max = maximum. Measurements with an asterisk are raw data.

| **Site** |  | **P2_L** | **P2_w** | **P3_L** | **P3_w** | **P4_L** | **P4_w** | **M1_L** | **M1_w** | **E_H** | **M2_L** | **M2_w** | **E_H** | **M3_L** | **M3_w** | **E_H** |
| --- | --- | --- | --- | --- | --- | --- | --- | --- | --- | --- | --- | --- | --- | --- | --- | --- |
| Teixoneres_IIb |  |  |  | 9,36* | 9,91* |  |  |  |  |  |  |  |  |  |  |  |
| Teixoneres_IIIa |  |  |  | 8,43* | 8,6* | 7,03* | 9,69* |  |  |  |  |  |  |  |  |  |
| Teixoneres_IIIb | n | *5* | *6* | *4* | *4* | *6* | *5* | *2* | *3* | *5* | *4* | *3* | *5* | 10,54* | 12,25* | 7,66* |
|  | m | **9,81** | **9,03** | **9,45** | **9,77** | **8,37** | **9,64** | **9,88** | **9,30** | **5,29** | **12,23** | **14,19** | **7,84** | 10,4* | 12,29* | 5,08* |
|  | s | 0,60 | 0,65 | 0,36 | 1,09 | 0,81 | 1,13 | 0,76 | 2,83 | 1,93 | 2,31 | 2,15 | 2,70 |  |  |  |
|  | min | 9,00 | 8,30 | 9,02 | 8,20 | 7,57 | 7,90 | 9,34 | 7,48 | 3,77 | 10,00 | 12,45 | 5,35 |  |  |  |
|  | max | 10,6 | 10,1 | 9,86 | 10,5 | 9,53 | 10,98 | 10,42 | 12,56 | 8 | 15,47 | 16,6 | 12,4 |  |  |  |
| Pié Lombard |  | 9,4* | 10,27* |  |  |  |  |  |  |  |  |  |  |  |  |  |
| **Site** |  | **p2_L** | **p2_w** | **p3_L** | **p3_w** | **p4_L** | **p4_w** | **m1_L** | **m1_w** | **E_H** | **m2_L** | **m2_w** | **E_H** | **m3_L** | **m3_w** | **E_H** |
| Teixoneres_IIIa |  |  |  |  |  | 10,35* | 7,67* | 11* | 7,87* | 5,33* |  |  |  | 16,01* | 8,16* | 6,66* |
| Teixoneres_IIIb | n | 6,34* | 4,16* | *7* | *7* | *3* | *3* |  | 7,47* | 6,06* | *3* | *4* | *5* | *4* | *4* | *4* |
|  | m | 6,6* | 3,2* | **10,50** | **7,41** | **10,54** | **7,14** |  |  | 3,1* | **11,37** | **8,12** | **6,26** | **16,56** | **8,33** | **8,73** |
|  | s |  |  | 1,72 | 1,63 | 0,54 | 0,31 | 11,54* | 7,8* | 9,54* | 0,53 | 0,28 | 1,72 | 3,24 | 2,53 | 1,83 |
|  | min |  |  | 8,52 | 5,24 | 9,92 | 6,80 |  |  |  | 10,97 | 7,90 | 4,14 | 13,82 | 6,90 | 6,25 |
|  | max |  |  | 13,80 | 10,00 | 10,90 | 7,41 |  |  |  | 11,97 | 8,50 | 8,88 | 21,26 | 12,11 | 10,63 |
| Pié Lombard |  |  |  | 8,44 | 5,37 | 9,00 | 5,60 |  |  |  |  |  |  | 16,71* | 8,22* | 8,69* |

Table S8: Summary of the measurements of the upper and lower permanent teeth of *Capreolus* from Teixoneres and Pié Lombard. L = length, w = width, E_H = height of the entostyle/ectostylid. n = sample size; m = mean; s = standard deviation; min = minimum; max = maximum. Measurements with an asterisk are raw data. All the measurements are taken at the base of the crown.

**Discussion**


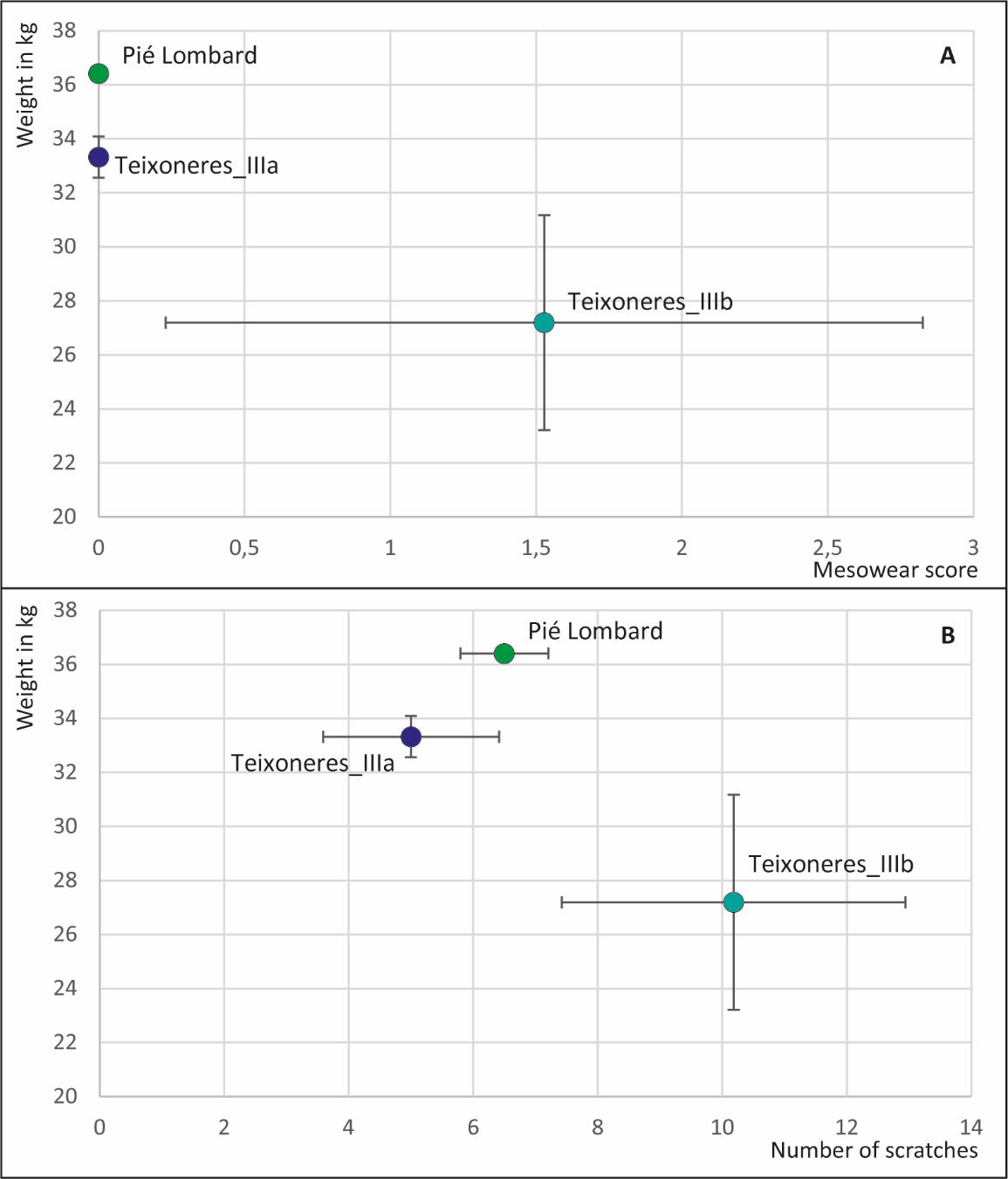


Fig. S5: Scatter plot comparing the estimated weight in kilograms of *C. capreolus* from Teixoneres unit IIIa and IIIb and Pié Lombard to: A: their mesowear score, B: their mean number of scratches.
